# Reactive and Additive Modifications of Styrenic Polymers with Phosphorus-Containing Compounds and Their Effects on Fire Retardance

**DOI:** 10.3390/molecules25173779

**Published:** 2020-08-19

**Authors:** Aloshy Baby, Svetlana Tretsiakova-McNally, Malavika Arun, Paul Joseph, Jianping Zhang

**Affiliations:** 1 Belfast School of Architecture and the Built Environment, Ulster University, Newtownabbey BT37 0QB, UK; baby-a1@ulster.ac.uk (A.B.); j.zhang@ulster.ac.uk (J.Z.); 2 Institute of Sustainable Industries and Liveable Cities, Victoria University, PO Box 14428, Melbourne 8001, Victoria, Australia; malavika.arun@live.vu.edu.au (M.A.); paul.joseph@vu.edu.au (P.J.)

**Keywords:** styrenic polymers, phosphorus containing fire retardants, fire retardance, thermal stability

## Abstract

Polystyrene, despite its high flammability, is widely used as a thermal insulation material for buildings, for food packaging, in electrical and automotive industries, etc. A number of modification routes have been explored to improve the fire retardance and boost the thermal stability of commercially important styrene-based polymeric products. The earlier strategies mostly involved the use of halogenated fire retardants. Nowadays, these compounds are considered to be persistent pollutants that are hazardous to public and environmental health. Many well-known halogen-based fire retardants, regardless of their chemical structures and modes of action, have been withdrawn from built environments in the European Union, USA, and Canada. This had triggered a growing research interest in, and an industrial demand for, halogen-free alternatives, which not only will reduce the flammability but also address toxicity and bioaccumulation issues. Among the possible options, phosphorus-containing compounds have received greater attention due to their excellent fire-retarding efficiencies and environmentally friendly attributes. Numerous reports were also published on reactive and additive modifications of polystyrene in different forms, particularly in the last decade; hence, the current article aims to provide a critical review of these publications. The authors mainly intend to focus on the chemistries of phosphorous compounds, with the P atom being in different chemical environments, used either as reactive, or additive, fire retardants in styrene-based materials. The chemical pathways and possible mechanisms behind the fire retardance are discussed in this review.

## 1. Introduction

Whilst many synthetic commodity plastics have excellent properties, such as light weight, good weatherability, and can be easily manufactured and/or processed into a variety of products with a range of useful applications, they also possess some obvious disadvantages [1]. One of them is high flammability, which often restricts their wider applicability, especially as the building materials, or as the components used in the transport sector [2]. Some chain-growth polymers can be easily ignited, leading to a fast and uncontrolled fire spread. Therefore, it is essential to improve the fire-retardant properties of these materials to warrant their safer and wider use in the construction and transport industries. This is often achieved by treating a polymeric material through a suitable methodology, where an appropriate combustion inhibitory reagent, a fire retardant (FR), is incorporated into the final product. A large number of FRs have been used for many years to protect polymers from the hazards of fire [1,3,4,5]. These compounds can be incorporated by physical means in the form of additives to the polymer matrix. Another way of improving fire retardance is to prepare the inherently less flammable polymers through *co-* or *ter-* polymerisations of relevant monomers with unsaturated compounds that can impart fire resistance [6].

**Figure 1:**
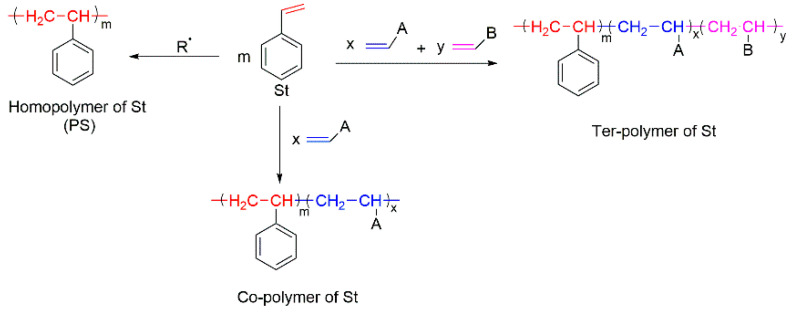
A general scheme of *homo*-, *co-,* and *ter-* polymerisation of styrene (R: is an initiator).

Polystyrene (PS) is a well-known thermoplastic polymer that is used worldwide for various applications owing to its low costs, ease of processing, excellent chemical resistance, low density, electrical/thermal insulation, etc. [7]. The styrenic polymers fall into different sub-classes [7,8,9,10,11,12,13,14]. Firstly, it is a homopolymer of styrene, which is also known as a general purpose polystyrene (GPPS) [7]. Secondly, the styrene monomer can be polymerised with other monomers to produce *co-* and/or *ter-* polymers, often having improved mechanical properties (Figure 1). The examples from this sub-class include styrene-acrylonitrile (SAN), acrylonitrile–butadiene-styrene (ABS), styrene–butadiene rubber (SBR), etc.

Furthermore, there are two types of polystyrene foams that are available commercially: expanded polystyrene (EPS) and extruded polystyrene (XPS) [8,9,10,14]. The EPS is a rigid closed-cell foam used for food packaging and thermal insulation [11,14]. The building sector also utilises the XPS, which is a closed-cell foam with higher density, better surface toughness, lower thermal conductivity, and higher stiffness compared to the EPS. The XPS foam is more suitable for weather exposure owing to its better water diffusion resistance compared to the EPS [7]. The EPS is typically manufactured from polystyrene beads with the aid of a blowing agent such as steam, or a low boiling point aliphatic hydrocarbon, e.g., *n-*pentane or *iso-*pentane. The grain-shaped beads are expanded up to 50 times of their original size to produce a rigid foam [14]. Meanwhile, the XPS is prepared through the expansion of a melt, containing a blowing agent and other additives including fire retardants (FRs), to improve the characteristics of the foam [8,9,10]. High-impact polystyrene (HIPS) has gained great industrial attention because of its good impact strength, high rigidity, and heat distortion resistance [12]. The HIPS, representing a two-phase system with rubber dispersed in a continuous PS matrix, is prepared by the polymerisation of styrene in the presence of rubber latex materials.

As mentioned earlier, even though styrene-based materials are used in various fields such as thermal insulation for buildings, in electrical and automotive industries, the high flammability of styrenic polymers limits their wider uses. When these polymers are exposed to an external heat source, they depolymerise easily with the release of numerous volatile products such as styrene monomers, dimers, trimers, and other hydrocarbons [13,14]. In addition, the styrenic polymers burn very rapidly, generating a significant amount of smoke [14]. Furthermore, they have a tendency to undergo a combustion process associated with a low char production, frequently resulting in the melt flow and melt dripping phenomena [14]. The various styrene-based products have to meet a set of stringent fire safety requirements prior to the use within buildings, and, thus, it became necessary to develop the efficient fire-retardant systems for their fire protection. 

Over the last 40 years, a wide range of halogenated products were developed and successfully used for different forms of polystyrene. The high effectiveness of halogenated FRs, especially those containing bromine (Br) and chlorine (Cl), and their relatively low costs, made these materials very attractive fire-protective solutions [14,15]. These FRs dominated the polymer industry in the past, but recently, their application became closely monitored, and it is regulated in many countries due to their toxicity, persistence, and bioaccumulation issues. For instance, the study conducted by Wemken et al. (2020) discovered the presence of different brominated FRs in the breast milk of first-time mothers living in Ireland [16]. Another work reported the high levels of brominated compounds found in the urban soils from the city of Melbourne, Australia [17]. Moreover, halogenated FRs can cause severe health and environmental problems owing to the release of dioxins and furans in the post-fire situations. As a result, in recent years, the wider use of halogen-based FRs has been very severely criticized, and several formulations were banned completely in the European Union (EU), USA, and Canada [18]. Meanwhile, a positive response to the regulatory rules applied to this class of FRs is evident. For example, the ban on the use of hexabromocyclododecane (HBCD) and penta-bromodiphenyl ether (BDE) resulted in the lower concentrations of these compounds detected in the breast milk [16].

In the last decade, numerous attempts have been made to develop the environmentally benign fire-retardant formulations suitable for polymeric products [1,14,19,20,21,22]. Among the proposed FR solutions, the compounds containing phosphorus (P) atom(s) are considered to be less toxic than the corresponding halogen-containing formulations [1,3,5]. Despite a wealth of P-containing formulations being developed to reduce the flammability of the chain-growth polymers, it remains challenging to design non-toxic, environmentally friendly yet efficient FRs for PS. The majority of the proposed halogen-free alternatives work, predominantly, in the condensed phase, which is a less useful mode of fire retardance for the polymers with a poor ability to generate char. Nevertheless, the P-bearing FRs can be very powerful in controlling combustion phenomena, at least the processes arising from the vapour phase inhibitory action [19,20]. A comprehensive review of FRs for styrenic polymers was carried out by Weil and Levchik [14] more than 10 years ago, and it was largely dedicated to the halogenated options. The current review focusses on the phosphorus-containing FRs, used either as the additive or reactive modifications, for styrene-based materials. The different classes of phosphorous FRs and their chemical interactions with PS chains are critically analysed. The mechanisms behind the fire retardance are also discussed in detail in this paper, when/if the relevant publications report on this aspect.

## 2. Combustion and Fire Retardance of Polymeric Materials: General Considerations

**Figure 2:**
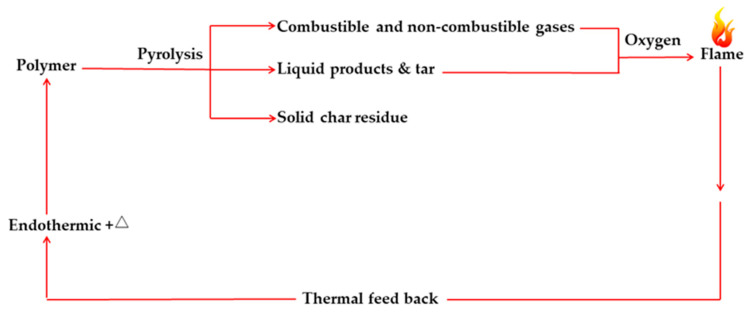
A diagram representation of a polymer combustion cycle.

Generally, polymer combustion processes can occur in the condensed phase, in the gaseous phase, and at the interphase [2] according to the combustion cycle shown in Figure 2. During the exposure to an external heat flux, a polymer can pyrolyse, generating the increased volumes of flammable volatiles released to the gaseous phase. Then, these volatile products mix with the atmospheric oxygen, forming a fuel source that is consequently ignited, leading to a flaming combustion. The heat generated during this process is fed back to the condensed phase of the polymer system, thus sustaining the burning process. Moreover, a tendency of the polymeric materials to melt and flow, forming a pool of flammable degradation products, can constitute a very serious secondary fire hazard, as it often can result in further ignition or the burning of surrounding fuel loads [23].

The commercial polymers exhibit a wide range of propensities to ignite. Generally, they require a temperature range of around 270–470 °C to undergo the ignition [24]. However, the EPS or XPS are highly ignitable even in the presence of low-intensity sources for piloted ignition, and they can initially go through a smouldering phase of combustion. The main reason behind this is a relatively high ignitability/flammability attributed to their porous structure [25,26,27,28,29]. 

The thermal decomposition pathways of the individual polymers are largely governed by their chemical structures [21]. Typical degradation patterns involve hydrogen transfer to α- or β-carbon atoms, oxygen or nitrogen atoms; cyclisation; side-chain reactions; molecular rearrangements; unzipping to a monomer; and the elimination of small molecules such as carbon dioxide, sulphur dioxide, or hydrogen sulphide [4,29,30]. Aromatic backbone-containing polymers may involve cross-linking and side-chain reactions leading to carbonaceous char residues [30]. In addition, the heat transfer to the condensed phase and the rate, at which the thermal energy is stored, are the important factors as they determine the time to ignition and the combustion rates of polymers [30]. Furthermore, the burning of solid polymer fuels generally depends on other environmental parameters such as pressure, temperature, an extent of oxygen ingression, as well as on the innate material properties associated with molecular, thermo-physical, thermo-chemical, and degradation characteristics. 

**Figure 3:**
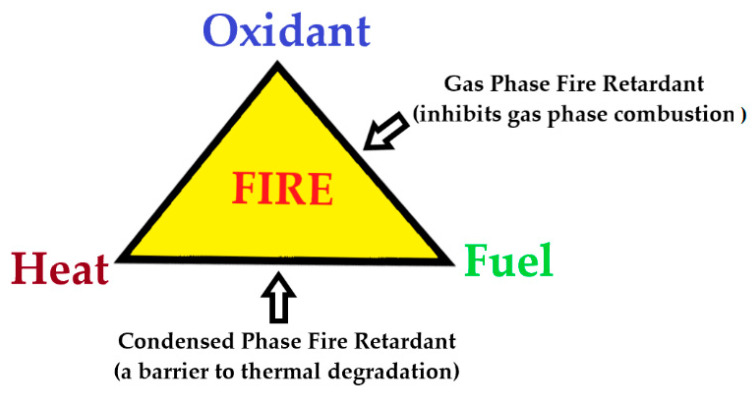
A diagram representation of fire-retardant (FR) actions interrupting a polymer combustion cycle.

The introduction of a FR into a polymer system can hinder certain stages of combustion, either in the condensed phase or in the gaseous phase (Figure 3).

In the condensed phase mechanism, the FR compound usually assists the formation of a carbonaceous char and/or an in situ production of water, while undergoing thermal decomposition. The char residue often generates a thick coating layer on a polymer surface, which prevents the release of flammable volatiles into the gaseous phase, thus obstructing this pyrolysis pathway. The intumescent coatings work by this principle as well [31,32]. It also helps protecting the underlying polymeric substrate from further thermal damages [33,34,35]. 

It is known that the combustion of a gaseous fuel mixture is facilitated by certain free radicals [33]. Most burning polymers generate free radicals, which then combine with the atmospheric oxygen [34,35]. Generally, H^●^ and HO^●^-centred radicals are the predominant intermediates that are responsible for the chain propagation reactions [1,30,33]. The HO^●^ species are also responsible for the secondary oxidation of carbon monoxide CO to carbon dioxide CO_2_ [30,34]. In most cases, the free radicals, formed by the burning polymer and released to the gaseous phase, are scavenged by the FRs, or by their degradation fragments, subsequently converting them into more stable species [34,35]. In turn, this interrupts the exothermic processes, leading to a less efficient combustion and a fire suppression. Most of the halogen-based flame retardants follow this mechanism of fire retardance [1].

The FRs can interact with the polymers physically and/or chemically depending on their inherent nature and the associated properties [4,34,35]. The predominant physical mechanisms include the following:

- A formation of a protective coating. This involves the formation of a carbonaceous char upon combustion, which then acts as a physical barrier between the polymeric substrate and the surrounding atmosphere. This limits the fuel supply to the system, thus hindering the combustion process. Such coatings also help to prevent the release of flammable volatiles/gases into the atmosphere.
- Cooling. During combustion, this type of FR activates certain endothermic reactions, which absorb the surrounding heat, thereby cooling the system below the temperature that is required to sustain the combustion process.
- Dilution. Flame spread can be hindered by adding certain inert additives, or fillers, to the polymer system. During flaming combustion, these additives liberate inert gases, which then dilute the system, making it less favourable for further burning in the gaseous phase.

Some FRs also control and limit the unwanted phenomena of melt-flowing and the melt-dripping in the polymeric systems [23,34]. 

## 3. Thermal Decomposition and Flammability of Styrenic Polymers

When ignited, PS and its copolymers can burn very fast with a visible flame, releasing volatiles into the environment including styrene monomer and the associated oligomers, benzene and lower alkylbenzenes, etc. [36,37,38]. During the burning process, PS can also melt flow and drip, which can lead to an increased fuel load, feeding into the enhanced flame spread [23,37]. Generally, styrenic polymers generate the minimal amounts of char residues, especially in oxygen-enriched atmospheres.

**Figure 4:**
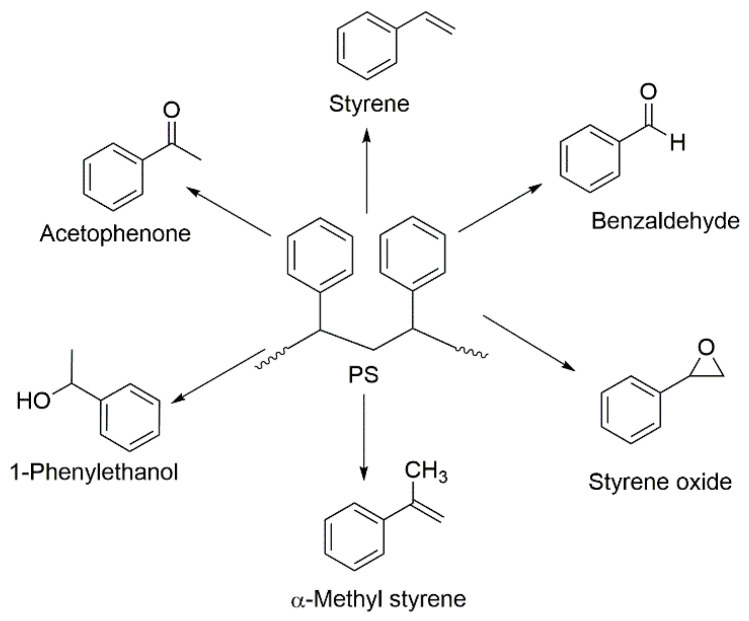
Thermal degradation products generated by burning polystyrene (PS) [36,38,39,40,41,42].

Generally, the styrene homopolymer starts decomposing at a temperature of around 270 °C and continues degrading until 425 °C under the normal conditions in the air [39]. Through the random main-chain cleavages and associated mechanisms, PS may form the varying amounts of styrene, benzaldehyde, styrene oxide, acetophenone, α-methyl styrene, and l-phenylethanol (Figure 4) [36,39,40]. Styrene and benzaldehyde are found to be the prominent fractions among all the degradation products [41,42]. 

Similar to the majority of main-chain carbon polymers, the thermal degradation of PS generally occurs in three steps: initiation, propagation, and termination, which follows a free-radical chain mechanism [36,42].

**Figure 5:**
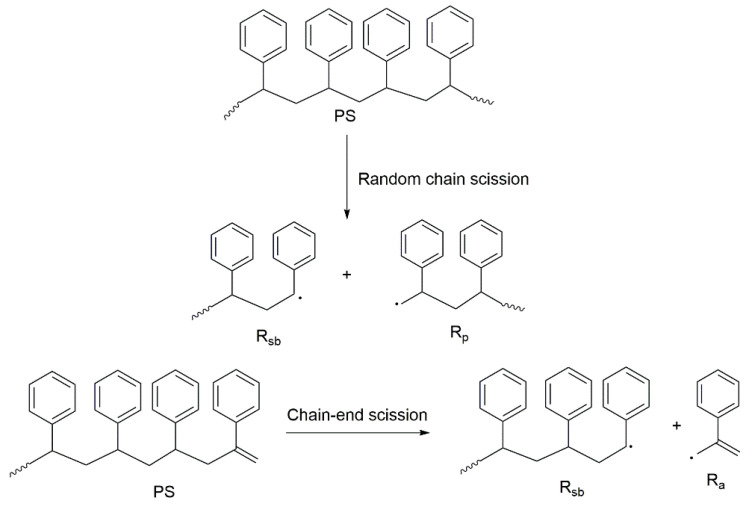
A schematic representation of initiation during the thermal decomposition of PS [36,42].

- Initiation
  The degradation of PS can take place via two routes, either through a random chain scission, or by a chain-end scission (Figure 5). In the case of the random chain scission, two radicals are formed, a primary radical (R_p_) and a secondary benzyl radical (R_sb_) with a strong benzylic resonance. In the chain-end scission mechanism, one secondary benzyl radical (R_sb_) and the resonantly stabilised allyl benzene radical (R_a_) are formed, as shown in Figure 5.
- Propagation
  The generated free radicals are propagated through hydrogen abstraction and unzipping reactions. There are two types of hydrogen abstraction reactions: intermolecular and intramolecular abstractions.
- Termination
  Termination can occur either by recombination or by the disproportionation mechanisms of various active radical fragments.

## 4. Fire Retardation of Styrenic Polymers with Phosphorous-Containing Compounds

In the past, HBCD and decabromodiphenyl oxide (DBDPO) were the most popular additives used in industry to improve the fire retardance of PS [14]. Hydrogen bromide, liberated over a temperature range specific for each of these formulations, acts as an effective free radicals scavenging agent during combustion. The negative impacts of halogenated formulations and hydrogen halides on the environment and human health prompted a search for more benign options [5,34]. The P-containing compounds, with lower toxicity levels, are as important as the halogenated FRs, and to a certain extent, they are as effective in controlling the combustion and flammability of PS [5,19,20,34].

A large number of P-containing compounds having different chemical environments of the P atom(s) are already available on the market [1,5,6,34]. These compounds, when thermally degraded, are shown to produce phosphoric acid and similar species that could inhibit the combustion process in the condensed phase [34,35]. Depending on the chemical nature of the phosphorus compounds, they can also be active in the gaseous phase: the PO^●^ radical can be considered as one of the most important species, acting as a scavenging agent [6,34,35,43]. Some common P-containing fire retardant compositions for the styrenic polymers include red phosphorus, phosphine oxides, inorganic phosphates, organophosphates, phosphonates, chlorophosphates, etc. [5,6,22,34]. Furthermore, some blends of P-containing compounds with other FRs exhibit a certain degree of synergism similar to the one observed for halogen/antimony combinations [2,5,6,34]. As phosphorus (P-) and nitrogen (N-) FRs, when combined, can also demonstrate synergism, the P/N-containing compounds, such as phosphorylaminoesters and phosphoramides, were found to enhance the polymers’ fire-retardant properties [21,44,45]. 

The P-containing compounds used as FRs for styrenic polymers include both additive and reactive types [1,6,21]. The efficiency, mode of action, advantages, and disadvantages of each type are well-documented and discussed elsewhere [1,5,6,34,35]. Generally, the organophosphorus compounds have good thermal and hydrolytic stabilities [1,5,6,21,34,35]. The efficiency of P-based compounds often depends on a number of factors: chemical environment and the oxidation state of the P atom; volatility; nature of the degradation products formed upon the thermolysis; etc. [6,34,35,46,47,48]. The condensed-phase activity of P-compounds within styrenic systems predominantly involves a char formation, which is facilitated by the dehydration of the polymeric structure, leading to cyclisation, cross-linking, aromatisation, and graphitisation [6]. The cross-linking can also be induced by the decomposition by-products of the phosphorus compounds. In the gaseous phase, these compounds act mainly by scavenging free radicals such as H^●^ and HO^●^, thus preventing further oxidation and decomposition of styrenic polymers [6,34]. 

Thus, it is clear that phosphorus-containing compounds can be as effective as, and in some cases, superior to many new or tried-and-tested FRs. Given the renewed interest in P-containing FRs, especially as the alternatives to environmentally harmful halogenated compounds, their desirable attributes are explored through the current review.

### 4.1. Additive Modifications of Styrenic Polymers

The most common route to an improved fire retardance is to incorporate the additive FRs during the polymers processing, which is usually achieved through the melt blending or solution/solvent-mixing techniques [49,50,51]. In the melt blending, the polymer is first melted and then combined with the corresponding FR using an extruder. This approach is very popular in industry because of its simplicity and the absence of noxious organic solvents. Meanwhile, the solution mixing begins with the dispersion of the additives in a polymeric solution followed by a controlled solvent evaporation and a composite film casting [52]. The organic and inorganic compounds, multi-component formulations, and a range of nanomaterials are used as the additive FRs for different sub-classes of styrenic polymers [53,54,55,56,57,58,59,60,61,62,63,64,65,66,67,68,69,70,71,72,73,74,75,76,77,78,79,80,81,82,83,84,85,86,87,88,89,90,91,92,93,94,95,96,97,98,99,100,101,102,103,104,105,106], and these are discussed in the following sections.

#### 4.1.1. Ammonium Polyphosphate (P^+5^)

Ammonium polyphosphate (APP) has gained great attention in developing FRs because of the high P- and N- contents. It has been extensively used for different polymers such as epoxy resins [53] and polyamides [54]. Additionally, APP is one of the main components of the intumescent fire-retarding systems, where it functions as an acid catalyst [55]. However, the individual use of APP as a FR for PS was not found to be effective, which is possibly due to the change in the morphology of char [56,57]. Hence, several attempts have been made to extend the utilisation of APP in PS, specifically by combining it with other agents. For example, Shi et al. (2016) utilised graphitic carbon nitride (g-C_3_N_4_) as an additive, along with APP, for enhancing the fire-retarding properties of PS [47]. The g-C_3_N_4_ has good chemical and thermal stability. The primary (-NH_2_) and secondary (-NH) amino-groups present in graphitic carbon nitride serve as the reactive sites either for hydrogen bonding or for reacting with other functional groups. In this study, g-C_3_N_4_ was treated with APP to prepare a FR formulation denoted as carbon nitride ammonium polyphosphate (CNAPP) by the solvent mixing method, followed by mixing with PS at a temperature of 180 °C [47]. Thermogravimetric analysis (TGA) proved that the thermal stability of the PS composite was enhanced by the addition of 20 wt% CNAPP. The maximum decomposition temperature (T_max_) of the PS/CNAPP composite was increased by 40 °C; meanwhile, the peak heat release rate (PHRR), measured through cone calorimetry (CC), was dropped by 65% compared to the neat PS [47]. The real-time infrared (RTIR) spectra revealed the presence of bands characteristic for P–O–C and P–N–C linkages, which were formed during the thermal oxidative degradation of the prepared composites. These cross-linked structures facilitated the formation of a char residue at higher temperatures (750 °C). Besides, g-C_3_N_4_, when present in the PS composites, accelerated the char formation, which resembled a vermiform structure that exhibited a “tortuous effect”. These char layers protected the PS polymer from further combustion and decomposition [47].

Recently, the syntheses of layered double hydroxides (LDHs) were carried out to obtain FRs suitable for the application in PS [58,59,60]. The LDH generally consists of positively charged metal hydroxide layers with interlayers of anions to maintain the overall charge neutrality [61]. Nyambo et al. (2008) prepared the fire-retarded PS by combining APP with a magnesium (Mg) and aluminium (Al)-based layered inorganic/organic hybrid, Mg–Al–undecenoate LDH (MAU-LDH) [56]. The purpose of using MAU-LDH as an additive FR along with APP is two-fold. Firstly, the combination of APP and LDH improves the dispersion of these additives within the polymer matrix, whilst the synergistic chemical interactions between APP and LDH allow reducing the amounts of FRs required to obtain a sufficient level of fire retardance. Secondly, the MAU-LDH works in the same way as the metal hydroxides, Mg(OH)_2_ and Al(OH)_3_, by slowing down the depolymerisation processes of PS, while at the same time forming water through dehydroxylation reactions [56]. This study demonstrated that the onset of thermal degradation (T_i_) for PS was shifted from 399 to 379 °C by the incorporation of 10 wt% APP alone, whereas the values of T_i_ were increased by nearly 10 °C as a response to the addition of 5 wt% MAU-LDH with 5 wt% APP. This result indicated the synergistic interactions of APP and MAU-LDH enhancing the thermal stabilisation of PS. However, the authors did not observe any significant increase in the char formation for PS-MAU/APP (5/5 wt%) composites [56]. 

**Figure 6:**
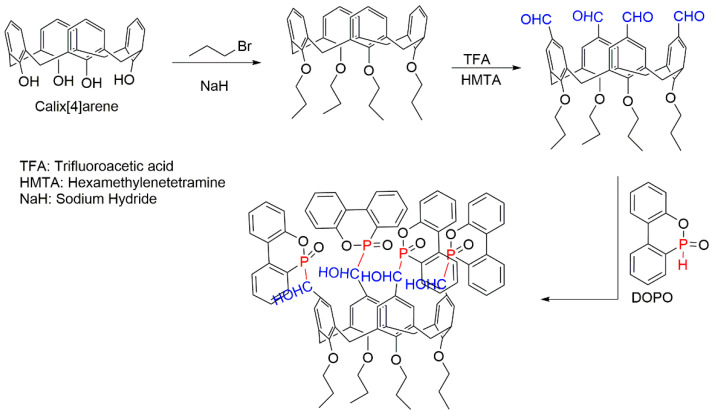
Synthesis of a novel FR based on calixarene and 9,10-dihydro-9-oxa-10-phosphaphenanthrene-10-oxide (DOPO) [62].

A novel formulation containing 9,10-dihydro-9-oxa-10-phosphaphenanthrene-10-oxide (DOPO) and calixarene was synthesised by Qian et al. (2018) as per the schemes shown in Figure 6 [62]. Subsequently, it was combined with APP and PS by the melt blending at 170 °C [62]. The total fire retardant loading was fixed at 30 wt% in all the cases. The char formation under the nitrogen atmosphere was increased dramatically from 0.79 wt% for the neat PS to 20.00 wt% for the composite with 15 wt% FR and 15 wt% APP. The fire-retardant effect was also clearly observed from the increased limiting oxygen index (LOI) values: 17 vol% for PS and 23 vol% for the modified PS composites [62]. The char layers formed in the condensed phase seemed not only to prevent the release of combustible gases but also to protect the interior surface of the polymer from the detrimental impact of heat. However, it is important to note here that none of these composites passed the vertical UL-94 burning test, even though during the UL-94 horizontal test all the composites exhibited a lower flame spread and no melt dripping compared to the unmodified counterpart. The results of all the tests carried out may indicate the improved thermo-oxidative resistance and fire retardance of the modified PS, which was due to the catalysing effects of APP on the char formation during combustion along with the synergistic effects between the FR and APP [62]. The APP was combined with clay to develop fire-retarded PS/Nylon-6 blends [63]. 

The dispersion and the compatibility of FRs with the polymer matrix are the important factors that affect the properties and fire-retarding efficiency of the final products. Ammonium polyphosphate is a hydrophilic inorganic material; hence, the dispersion of any hydrophobic FR with APP would affect the preparation, uniformity, and properties of the obtained composites. To overcome this difficulty, Ding et al. (2019) proposed the surface modification of APP that would improve its dispersion and compatibility with hydrophobic carboxylic-functionalised multi-walled carbon nanotubes (COOH-MWCNTs) [64]. The surface of APP was treated with a silane-coupling agent, 3-aminopropyl triethoxysilane (KH 550), to obtain the modified APP (k-APP). The HIPS nanocomposites were prepared by the melt blending with k-APP and COOH-MWCNTs. The scanning electron microscopy (SEM) micrographs revealed an enhanced dispersion of k-APP in the HIPS matrix that also seemed to prevent the agglomerations of the suspended FR particles compared with the unmodified APP. The uniform distribution of FRs within the polymer matrix was directly reflected in better mechanical properties of the polymers. Indeed, the HIPS composites with 15 wt% k-APP were characterised by the 5.0–7.0% increase in flexural modulus and tensile strength, when compared with those containing the unmodified APP. As opposed to the neat HIPS, the synergistic effect of k-APP and COOH-MWCNTs improved the thermal stability and fire retardance of the composites by introducing 12 wt% k-APP/3 wt% COOH-MWCNTs. This was evident from the increase in the values of T_i_ by 13 °C and T_max_ by 16 °C, from the 681 kW/m^2^ drop in the PHRR, and from the 0.30 kg/kg reduction in CO yield. Similarly, nano-sized oxides such as alumina and silica, treated with octylsilane in combination with APP, were used to impart the fire resistance to polymethylmethacrylate (PMMA) and PS [65]. This comparative study revealed that hydrophobic nano-oxides with APP have a more positive impact on the thermal stability and fire retardance to PS than the hydrophilic oxides mixed with APP [65]. 

#### 4.1.2. Aluminium Hypophosphite (P^+1^)

Aluminium hypophosphite (AP) belongs to another category of P-containing inorganic fire retardants. AP has been widely used in various polymers because of its excellent fire-retarding performance, cost effectiveness, and environmental friendliness. Initially, AP was incorporated as a single additive to PS composites by melt blending [66]. The V-0 rating in the UL-94 test was achieved in the composites containing high amounts of AP, between 25 and 30 wt%. As a result of this modification, the char residues were increased dramatically, from 0.29 wt% for the neat PS to 16.00 wt% and to 24.00 wt% for the composites with 25 wt% and 30 wt% of AP, respectively. Notably, the char residue formed in the air was approximately 4 wt% higher than that formed under the nitrogen atmosphere for all the PS/AP composites, which was attributed to the formation of oxidised products of AP during thermal degradation of the polymeric materials [66]. 

**Figure 7:**
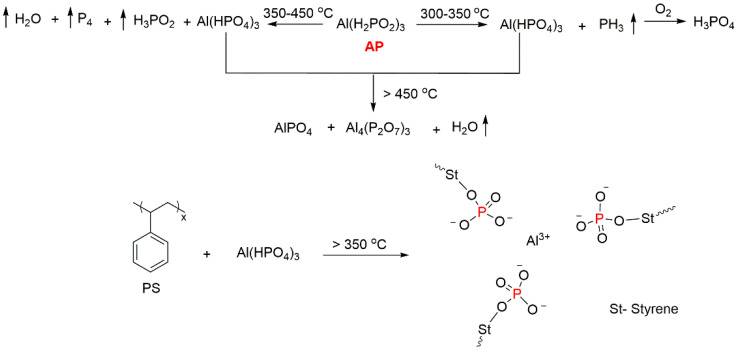
Fire-retarding mechanisms occurring within PS/AP (aluminium hypophosphite) composites [66].

The studies of fire-retarding mechanisms in PS/AP composites revealed the combined gaseous phase and condensed phase activity of AP during combustion [66]. The investigation of the decomposition processes of PS/AP composites indicated that during combustion, AP decomposed prior to PS, and the pyrolysis products such as phosphine (PH_3_), hypophosphorus acid (H_3_PO_2_), and red phosphorus (P_4_) were released. Among these products, PH_3_ can be further oxidised to generate phosphoric acid (H_3_PO_4_), which can promote charring (Figure 7). The formed P–O cross-linked structure acts as an insulating layer, which can also cover the polymer surface and protect it from fire. Meanwhile, in the gaseous phase, phosphinate ions (PO_2_^−^) formed during combustion seemed to prevent the flame growth and further development of the fire front [66].

The latest reports describe the processes when AP was added along with other compounds, with the purpose of lowering the loadings of the FR and to enhance the fire-retarding efficiency [67,68,69,70,71]. The study conducted by Zhu et al. (2018) described the incorporation of expandable graphite (EG) in combination with AP for preparing fire-retarded styrenic composites, with the AP/EG content being fixed at 15 wt% [67]. The UL-94 vertical burning tests revealed that that the polymers containing either 15 wt% of AP or 15 wt% of EG failed them, whereas the PS with 10 wt% of EG and 5 wt% of AP (PS/10EG/5AP) passed these tests, achieving the V-0 rating. In addition, this modification resulted in the reduction of the PHRR, measured through the CC, from 730 kW/m^2^ for PS to 163 kW/m^2^ for the PS/EG/AP composite. The extent of char formation also increased by up to 12 wt% for the modified styrenic polymers [67]. The enhanced fire retardance of the composites can be attributed to both the condensed phase and the gaseous phase activities of EG and AP. In the condensed phase, EG expanded to form a ‘worm’-like char residue, which turned out to be not compact enough to protect the PS surface from combustion. In comparison, the pyrophosphates generated from the decomposition of AP covered the polymer surface, resulting in a more compact char structure, which protected the inner polymer matrix from further decomposition. Meanwhile, in the gaseous phase, AP tends to form several P-containing substances that, in turn, were able to generate phosphorus radicals. These radicals captured highly reactive free radicals formed during the polymer combustion and thus effectively inhibited fire growth [67]. 

**Figure 8:**
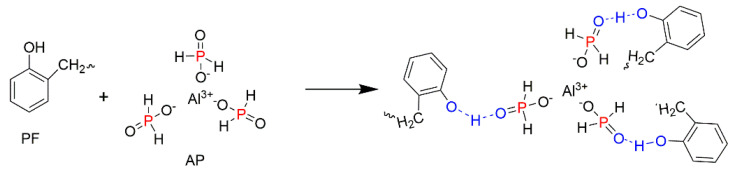
An interaction of phenolic resin (PF) with AP through hydrogen bonding in the PF/AP formulation [68].

In another work, a coating based on a mixture of AP with thermoplastic phenolic resin (PF) was prepared for the protection of the EPS [68]. The performance of EPS/PF/AP was compared with the one of the EPS containing PF and EG (EPS/PF/EG). It was reported that PF interacted with AP through the hydrogen bonding, as shown in Figure 8 [68]. More drastic reduction in the flammability and smoke release rate was observed for the EPS treated with the PF/AP pair compared to the material with added PF/EG. Thermal conductivity is one of the important properties that determines whether the material can be used for thermal insulation purposes. Interestingly, the thermal conductivity of the modified EPS foam remained at a very low level and changed only slightly, from 0.031 W/m∙K for the neat EPS to 0.038 W/m∙K for the EPS/PF/AP [68]. At the same time, the compressive strength more than doubled from 95 kPa for the EPS to 204 kPa for the EPS coated with PF/AP [68]. Thus, the EPS/PF/AP has a potential to be used as a suitable thermal insulation material. 

**Figure 9:**
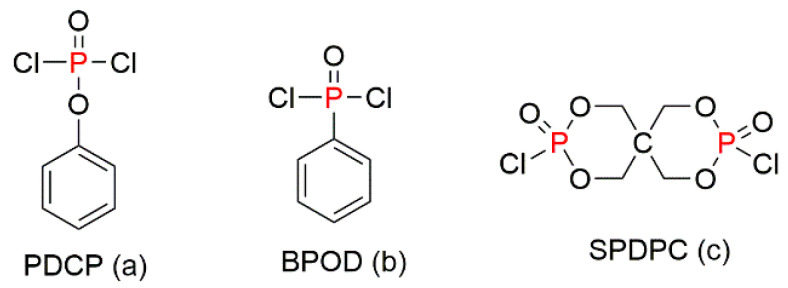
Chemical structures of phosphorous compounds used for the modification of g–C_3_N_4_ [70,71].

The aluminium hypophosphite can also be used with a two-dimensional material such as carbon nitride (g-C_3_N_4_), which was mentioned earlier. Zhu et al. (2017) proposed the modification of g-C_3_N_4_ with diethylphosphinic acid oligomers through hydrogen bonding, which was followed by the synthesis of a g-C_3_N_4_/organic aluminium diethylhypophosphite hybrid (g-C_3_N_4_/DAHPi) to improve not only the fire retardance but also the distribution of the graphitic material in the polymer matrix [69]. Similarly, g-C_3_N_4_–organic aluminium hypophosphite (g-C_3_N_4_/OAHPi) hybrid was prepared using two different phosphorus compounds: phenyldichlorophosphate (PDCP, Figure 9a) and bicholorophenylphosphine oxide (BPOD, Figure 9b) [70]. Furthermore, a novel two-step synthetic approach has been adopted to prepare layered organic aluminium hypophosphonate (OAHPi) [71]. Spirocyclic pentaerythritol bisphosphorate disphosphoryl chloride (SPDPC, Figure 9c) was used as the component for interacting with AP; then, it was incorporated into PS via the melt blending. This layered component provided a thermal barrier effect for the entire polymer matrix. It is important to note that all organically modified APs were characterised by enhanced thermal stability, and better fire safety was achieved at very low loadings of FRs (from approximately 0.5 to 5 wt%) by the combined gaseous and condensed phase mechanisms [71].

#### 4.1.3. Phosphorus-Containing Intumescent Formulations (P^+5^)

The intumescent fire retardants (IFRs) have been used for a long time for fire protecting a variety of materials and surfaces. Typically, an IFR system consists of an acid source (APP, phosphoric acid, etc.), a char-forming agent such as pentaerythritol (PER), and a blowing agent (e.g., melamine (MEL), or polyamides) [72]. When the IFR is exposed to fire, the charring and acid agents trigger the formation of a charred layer followed by its growth and expansion with the aid of a blowing agent. The expanded carbonised layer generated on the surface of a polymeric substrate acts as an insulating barrier, not only preventing the heat transfer between the heat source and the surface, but also limiting the fuel transfer and the diffusion of oxygen into the material [72,73]. 

Lu and Wilkie (2010) used APP and tripentaerythritol (TPE) as an acid source and carbonisation agents, respectively, for developing an IFR system [74]. The PS/IFR composites were prepared by melt blending at 180 °C with 20 wt% of the IFR in the final material. A partial substitution of the IFR with the small amounts (2 wt%) of different nanofillers produced the PS/IFR composites containing montmorillonite (MMT) clay, MWCNTs, iron oxide (Fe_2_O_3_), nickel (Ni) catalyst, and zirconium (Zr) phosphate. Thermal studies demonstrated that the composites with MMT clay and Ni catalyst exhibited the highest efficiency: the values of the PHRR were reduced by almost 70% compared to the unmodified PS [74]. The CC results proved that the flammability of the PS/IFR composites was dependent on the nature of the nanofiller and was reduced in the following order: MMT clay > Ni catalyst > Fe_2_O_3_ > MWCNTs > Zr phosphates. This pattern was in agreement with the results obtained from TGA, particularly in terms of the char yields generated by each composite [74]. The higher fire retardance of the PS composite with MMT clay and Ni catalyst can be explained by the formation of an aluminophosphate structure and the catalytic effect of Ni in the cross-linking and carbonisation processes.

**Figure 10:**
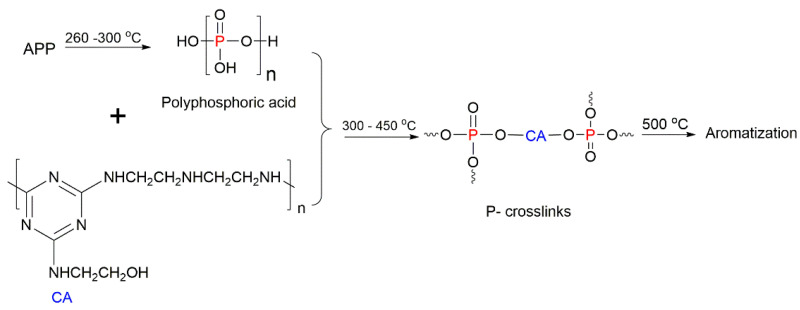
A char-forming mechanism in the ammonium polyphosphate/carbonisation agent (APP/CA) system [75].

Similarly, APP was used as an acid source along with a novel carbonisation agent (CA) triazin-2-aminoethanol diethylenetriamine in the IFR added to the polymers in the amount of 30 wt% [75]. The flammability studies demonstrated that the composite with a 3:1 weight ratio of APP to CA exhibited better efficiency than the materials with 1:1 and 2:1 weight ratios of APP:CA. The CC results for the PS/APP/CA (APP:CA = 3:1) composite revealed that the time to ignition (TTI) was decreased from 52 s to 31 s measured for the untreated PS [75]. It is a common trend that the TTI values of fire-retarded polymers are lower than those of the unmodified versions, which is associated with the decomposition of the added FRs during the initial stages of combustion. The synergistic effect between APP and CA resulted in the improved fire retardance of the composite styrenic materials, which was gauged from the increase in the T_max_ by 16 °C; better char production (up to 17 wt%); the decrease by 938 kW/m^2^ in the PHRR, and the lower total heat release (40 MJ/m^2^) compared to the neat PS [75]. In addition, the smoke production rate (SPR) was reduced from 0.254 m^2^ ∙s^−1^ for PS to 0.008 m^2^∙s^−1^ for the PS/APP/CA composite, whilst the CO emission decreased by 88% [75]. These changes can be explained by the decomposition of APP and a formation of polyphosphoric acid, which reacts with the molecules of triazin-2-aminoethanol diethylenetriamine, eventually promoting the dehydration processes between OH groups present in the CA (Figure 10). The obtained P–O–C cross-links are transformed further, at relatively high temperatures, through the aromatisation, producing P-rich charring residues, thus having a positive impact on the fire retardance as well as on the thermal stability of the composites [75]. Despite the significant improvement in the fire retardance of the modified styrenic composites, this system requires relatively high loading of the IFR (30 wt%), which negatively affected the mechanical properties of the protected polymers. Indeed, the tensile strength was reduced from 43.5 MPa for the unmodified PS to 30.5 MPa for the PS/APP/CA composite, whilst the values of flexural strength dropped from 71.2 MPa in PS to 55.5 MPa in the modified polymer. Meanwhile, there was no influence on the impact strength of the PS/APP/CA composite [75].

Another P-containing IFR system is based on a combination of zeolites, APP and PER, with the addition of a Lewis acid as a char promoter [76]. In this study, 4A-zeolite was treated with ammonium chloride (NH_4_Cl), to obtain an NH_4_^+^-exchanged zeolite (4A-NH_4_^+^), which was then heated to 550 °C and 770 °C to obtain the Lewis acid (4A-L zeolite) and the Bronsted acid (4A-H^+^ zeolite), respectively. It was found that the PS composite with 18 wt% of APP and 2 wt% of 4A-L zeolite had a higher efficiency in the char formation and better fire retardance in comparison to the composite containing 4A-H^+^ zeolite. The Lewis acid based on 4A-L zeolite can catalyse the cross-linking reactions within the PS/APP/4A-L composite. The water generated during the esterification of APP and PER interacted with the Lewis acid on the 4A-L zeolite, producing different complex compounds. Then, these complexes continuously reacted with the unsaturated -C=C- bonds to form primary carbonium ions, which resulted in the acidic catalysis, Friedel–Crafts alkylation, and a char formation [76]. 

**Figure 11:**
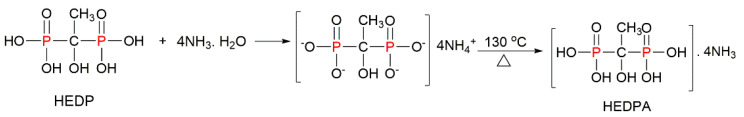
A synthetic route for the preparation of ammonium salt of HEDPA [77].

In another study, Xia et al. (2014) advocated the use of ammonium salts of 1-hydroxy ethylidene-1,1-diphosphonic acid (HEDPA) as an acid source in the IFR formulations [77]. The performance of the composites containing HEDPA was compared with those containing the precursor, 1-hydroxy ethylidene-1,1-diphosphonic acid (HEDP) (Figure 11). It was demonstrated that the efficiency of the materials with the salt (HEDPA) moieties is far superior to that observed for the PS composites with the acid (HEDP), since the former have achieved the V-0 rating in the UL-94 testing and the LOI increased to from 17 vol% 28 vol% measured for the unmodified PS [77]. Furthermore, the new IFR system with HEDPA/PER/MEL exhibited an excellent synergistic effect and a good compatibility with the PS matrix [77].

#### 4.1.4. Transition Metal-Based Formulations (P^+5^)

The transition metal-based organophosphorus compounds are an emerging class of hybrid FRs suitable for the fire protection of styrenic polymers. Recently, Wang et al. (2018) applied organophosphorus hybrid phenyphosphinates (PPIs) of different transition metals to enhance the fire-retarding properties of PS [78]. The phenyl groups bonded to phosphorus atoms within the PPI were located above and below the plane containing the metal atoms. This arrangement resulted in the formation of a conjugation effect between the PS and PPI, which helped obtain better dispersion in the polymer matrix and enhance the thermal barrier action. Additionally, these formulations not only favoured the char formation but also suppressed the yields of smoke and toxic volatiles emitted during degradation of the polymers. For instance, the PPI containing transition metals such as cobalt (Co), nickel (Ni), iron (Fe), and lanthanum (La) were synthesised and added to the PS via the solution-blending method [78]. The metal PPI, which is well-dispersed in the polymer matrix, seemed to obstruct the transfer of degraded products to the gaseous phase, and the transition metal appeared to catalyse the conversion of attached fragments to the char. Moreover, the addition of these metal PPIs suppressed the heat release, and it also reduced the smoke release rate and the CO emissions [78]. In the similar way, layered metal phenylphosphonates (PP) have been synthesised through a hydrothermal method [79]. The prepared Co–PP, Ni–PP, copper (Cu)–PP, and Fe–PP were added to the PS via the solution-blending approach. The fire-retarding mechanism was found to be similar to the one occurring in metal–PPIs. It was explained by the fact that the layered PP acted as a barrier, which in turn decreased the thermal decomposition rate of the PS composites and limited the gas diffusion [79]. It is important to mention that the main drawback of high loadings common for the additive FRs was mitigated here: the transition metal organophosphorus compounds were used in the amounts ranging from 2 to 6 wt% [79]. Thus, the formulations based on the transition metal PP and/or PPI are the promising candidates for enhancing the fire retardance of PS. 

#### 4.1.5. Functionalisation of Inorganic FRs with Organophosphorus Compounds (P^+5^)

**Figure 12:**
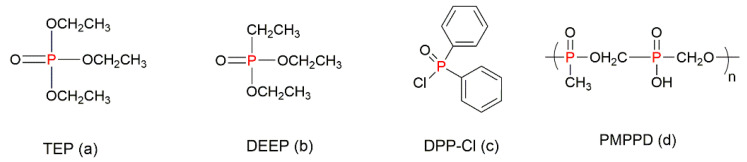
Chemical structures of organophosphorus additive FRs [38,80,84].

The additive modification of styrenic polymers with organophosphorus compounds can be achieved either by using them individually, or by the functionalisation, and/or in combination with other fire-retarding materials. For example, Price et al. (2007) used triethylphosphate (TEP, Figure 12a) and diethylethylphosphonate (DEEP, Figure 12b) through the additive incorporation into the PS systems [38]. The phosphorus content in these compounds was not higher than 3.5 wt%. The fire-retarding mechanism was reported to occur in the gaseous phase, regardless of whether the compound was phosphate (TEP) or phosphonate (DEEP) [38]. 

Furthermore, several organophosphorus compounds were used as additives in combinations with inorganics or nanomaterials. For instance, a nucleophilic substitution reaction of diphenylphosphinic chloride (DPP-Cl, Figure 12c) with an aminated multi-walled carbon nanotube (A-MWCNT) had led to the formation of a P–N-containing intermediate (DPPA-MWCNT) that was consequently mixed with PS via the melt blending at 190 °C [80]. The modification of PS with 1 wt% DPPA-MWCNT increased its fire retardance that was evident from the reduction in the toxic emissions such as smoke, CO, and CO_2_. Meanwhile, the PHRR values dropped from 907 kW/m^2^ for the neat PS to 625 kW/m^2^ for the PS/DPPA-MWCNT [80]. The enhanced fire retardance level was attributed to the barrier effect of carbon nanotubes (CNTs), which might provide sufficient time for the functionalised P-containing FR to trap the polymeric radicals, thereby inhibiting the further thermal degradation of PS and also leading to better char formation. Even though the CNT was functionalised with P and N groups, the synergistic effect of those two atoms was not clearly explained by the researchers [80]. In a similar way, the P-functionalised P-MWCNTs were prepared by treating the oxidised MWCNTs with phosphoric acid followed by the incorporation into PS via the solution blending [81]. The incorporation of 2 wt% of P-MWCNT into the polymer resulted in the 27% decrease of the total heat release (THR), while doubling the amount of char residue formed [81].

Graphene oxide (GO) is commonly used as the additive FR for styrenic polymers [50,82,83]. The functionalisation of GO with organophosphorus compounds can boost the thermal properties of the polymer composites. For instance, Qiu et al. (2015) synthesised the organophosphorus oligomer, poly(methylphosphonylbis(hydroxymethyl)hypophosphate) (PMPPD, Figure 12d), for the preparation of the functionalised graphene oxide (FGO) [84]. The incorporation of 5 wt% of FGO into PS resulted in the reduction of the heat release rate by 38%, while the value of T_max_ increased by 20 °C [84]. Along with better fire protection, the mechanical properties of the polymer such as its tensile and impact strengths were also improved due to the synergism between the PMPPD and FGO sheets. However, both the tensile and the impact strength of the PS/FGO composites were reduced when the loading of FGO grew from 0.1 wt% to 5 wt% [84]. Similarly, the functional modification of GO was carried out with the aid of DOPO to obtain the fire-retardant PS composite microspheres [85]. The gaseous phase free radical quenching of the functionalised DOPO and the condensed phase catalytic carbonisation effect of the GO sheets contributed to the overall enhancement of the fire safety of the polymer [85].

**Figure 13:**
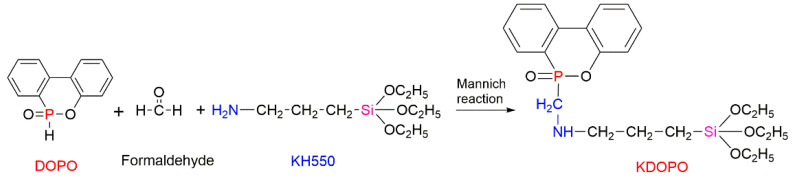
A synthetic route for the preparation of the KDOPO FR formulation [86]. KDOPO: Mannich reaction between the 9,10-dihydro-9-oxa-10-phosphaphenanthrene-10-oxide (DOPO) derivative and γ-aminopropyltriethoxysilane (KH550).

The Mannich reaction between the DOPO derivative and γ-aminopropyltriethoxysilane (KH550) resulted in the formation of the FR formulation, KDOPO, containing P, N, and Silicon (Si) atoms (Figure 13) [86]. The silanisation reaction between KDOPO and GO produced the functionalised GO, labelled as DGO, which was consequently applied onto the surface of the EPS through the simple surface-coating method. The modified foam exhibited better fire-retarding performance: the LOI values were up by 29%, and the V-0 rating (UL-94 test) was achieved by the addition of 20 g of the DGO to the EPS. In this study, it was shown that after the addition of DGO, the values of thermal conductivity were increased by 6.8%, although this is not sufficient to alter the thermal insulation property of the EPS [86]. The flammability studies revealed that each element within the FR composition, i.e., P, N, and Si, has a well-defined role to play in the fire-retarding mechanism. The P-containing fragment of the FR structure functioned in both the gaseous and the condensed phases, and the Si-containing group generated a positive silica layer on the polymer surface to protect the polymer matrix, thereby boosting the condensed phase mechanism. Meanwhile, the N-containing part in the structure of DGO supported the gaseous phase mode of action through the release of inert gases, diluting the combustible products and reducing the heat from the fire [86].

**Figure 14:**
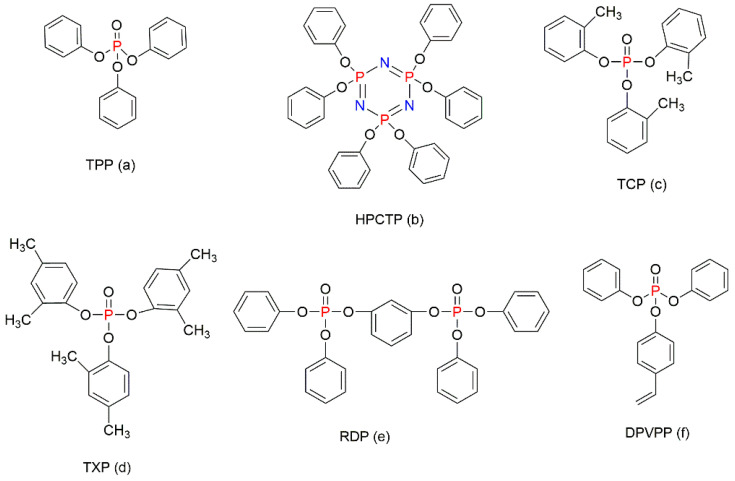
Chemical structures of different P-containing additive FRs [87,88,89].

The P-containing compounds such as triphenylphosphate (TPP) (Figure 14a) in the amount of 3 wt% and hexaphenoxycyclotriphosphazene (HPCTP) (Figure 14b), at 3 wt%, were used in combination with 25 wt% of EG and 25 wt% of melamine phosphate (MP) to prepare the foams by a supercritical carbon dioxide foaming [87]. As a result of this modification, the thermal stability and fire retardance of PS foams increased in the presence of TPP or HPCTP within the composition of the formulation. As for the thermal conductivity of the foam, its value was reduced from 0.0435 W/m∙K in the neat PS to 0.0372 W/m∙K and 0.0363 W/m∙K in the samples of PS foam with TPP and HPCTP, respectively [87]. The fire-retarding mechanism revealed that the highly viscous phosphate-based products formed during the degradation of MP were able to cover the surface of the EG, which further prevented its oxidation and generated a thick char layer. At the same time, TPP and HPCTP acted as synergists, thus enhancing the fire retardance of the modified styrenic material. The thermal decomposition of these compounds generated different P-centred radicals, which then quenched down other free radicals, thus inhibiting the combustion process [87]. 

Chigwada et al. (2003) evaluated a series of phosphate compounds to identify the best fire-retardant solution for the PS/nanoclay composite [88]. From the analysis, the phosphorus compounds such as tricesylphosphate (TCP, Figure 14c), trixylylphosphate (TXP, Figure 14d), and resorcinoldiphosphate (RDP, Figure 14e) were determined as the suitable fire retardants [88]. The PS nanocomposites with clay (1–10%) and phosphates (5–25%) were prepared by bulk polymerisation to achieve the fire retardance through synergy between these conventional phosphorus FRs and nano-sized additives [88]. In another study, 55% and 75% of diphenyl-4-vinylphenylphosphate (DPVPP) (Figure 14f) were used to modify clay that was then used as the additive FR for PS [89]. Even though the flammability of PS was decreased by this type of modification, high levels of FR loadings negatively affected the mechanical and other useful properties of PS [89].

**Figure 15:**
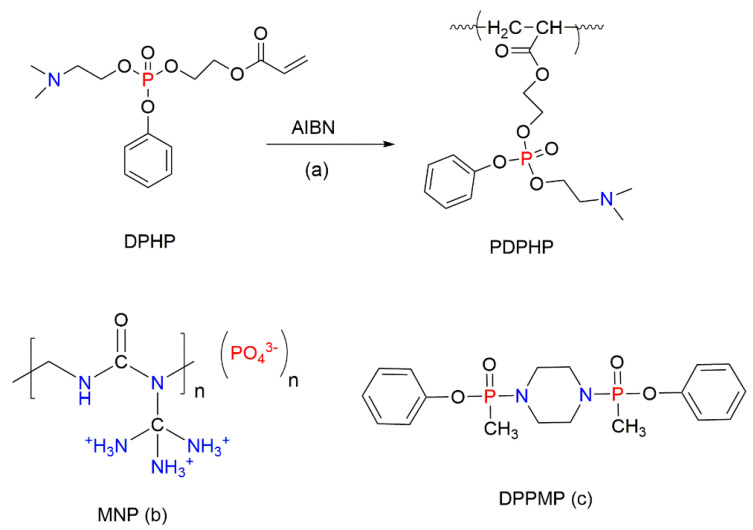
Synthesis of PDPHP oligomer (**a**), chemical structures of macromolecular P–N-based FR, (MNP) (**b**) and diphenyl piperazine-1,4-di-ylbis(methylphosphinate) (DPPMP) (**c**) [92,93,94]. PDPHP: oligomer of 2-dimethylaminoethylphenylhydroxyethylacrylate phosphate.

As mentioned earlier, the effective strategy to improve the fire retardance of styrenic polymers adopted by many researchers is to employ the P-containing formulations, which act in both the gaseous phase and in the condensed phase. This improvement is often achieved through the specific interactions of some elements such as phosphorous–nitrogen (P–N), phosphorous–silicon (P–Si), phosphorous–nitrogen–silicon (P–N–Si), and phosphorous–nitrogen–sulphur (P–N–S). The interactions of P-containing FRs with N-bearing compounds can be positive or negative, resulting in either synergistic or antagonistic effects, respectively [90]. The use of P–N synergistic FRs can exhibit the condensed phase mechanism of fire retardance, thus generating more carbonaceous char, which acts as a physical barrier and stops the further degradation of a polymeric material. Additionally, P–N-containing FRs can prevent the release of flammable and toxic volatile products [91]. However, some of the P–N-containing FRs are small molecular compounds that may have a relatively poor compatibility with the polymer matrix and hence could leach out very easily from the polymer composite over time. These problems can be solved through the use of oligomeric or polymeric FRs. For example, Tai et al. (2012) synthesised the P–N-containing compounds, 2-dimethylaminoethylphenylhydroxyethylacrylate phosphate (DPHP) and its oligomer PDPHP (Figure 15a) [92]. The T_max_ values increased from 433 °C in the neat PS to 446 °C in the PS composite with the added 30 wt% of PDPHP, while the char yield, recorded by TGA, was raised to 8.7 wt%. The flammability studies suggested that the enhancement in the fire retardance is caused by the gaseous and condensed phase actions. A macromolecular P–N-based FR, noted as MNP in Figure 15b, has been developed for the protection of the EPS foam [93]. The condensed and gaseous phase capabilities of MNP were responsible for better fire retardance of the modified foam. Even though the density of the foam almost doubled as a result of this compound being added, it did not affect the thermal conductivity of the EPS containing 30 wt% MNP. Meanwhile, the compressive strength of the modified foam was increased by 15%. Nguyen et al. (2008) carried out a comparative study of four different polymers with the added FR based on diphenyl piperazine-1,4-di-ylbis(methylphosphinate) (DPPMP, Figure 15c) [94]. It was found that DPPMP is not suitable for the polymers such as ABS, while it was very effective for protecting charring polymers, for example, polycarbonate and polybutylene terephthalate. The DPPMP additives imparted the gaseous phase fire retardance due to the presence of the CH_3_–P(=O)-unit [94]. 

**Figure 16:**
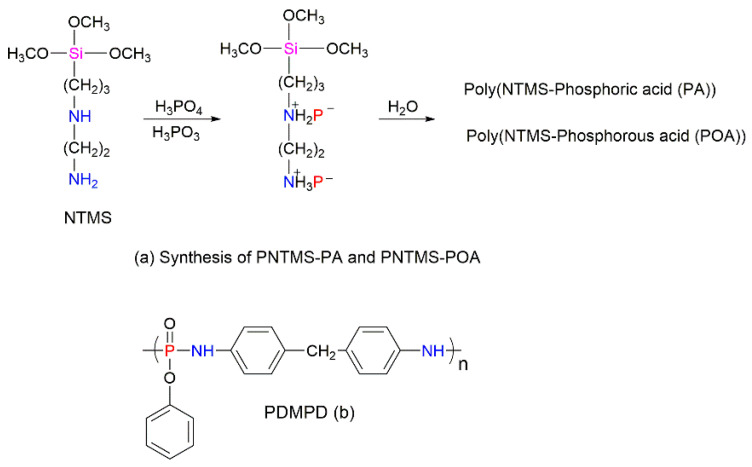
Synthesis of PNMTS-PA and PNTMS-POA (a) and the chemical structure of PDMPD (b) [95,97]. NMTS: *N*-β-(aminoethyl)-γ-aminopropyltrimethoxysilane, PA: phosphoric acid, H_3_PO_4_, POA: phosphorus acid, H_3_PO_3_, PDMPD: poly(4,4′-diaminodiphenylmethanephenyldichlorophosphate).

Some research groups used other elements such as Si or boron (B) to improve the efficiency of P–N-containing FRs further [95,96,97]. The addition of these compounds can endow the synergistic effect and thereby increase the charring and enhance the thermal performances of the polymers. For example, a novel FR containing P, N, and Si was developed from *N*-β-(aminoethyl)-γ-aminopropyltrimethoxysilane (NTMS, Figure 16a) reacted with phosphorus acid, H_3_PO_3_ (POA), and phosphoric acid, H_3_PO_4_ (PA), respectively, for incorporation into the EPS foam [95]. The obtained formulations were applied to the EPS by surface coating, which did not alter the key physical properties (e.g., density and compressive strength) of the foam. However, in this study, the synergism did not lead to any reduction in the amount of FR required to achieve the desired fire performance. Indeed, the lowest loading of the FR needed for the EPS to attain the V-0 rating in the UL-94 test remained relatively high, around 40 wt% [95]. Similarly, different B-containing compounds have been used as the synergistic FR additives to enhance fire safety of a range of polymers, including PS [96]. In the study carried out by Tai et al. (2012), zinc borate, 2ZnO∙3B_2_O_3_∙3.5H_2_O (ZnB), and boron phosphate, BPO_4_ (BP) were used as the synergists of poly(4,4′-diaminodiphenylmethanephenyldichlorophosphate) (PDMPD, Figure 16b) [97]. Approximately 2 wt% of BP and ZnB were added separately to the PS composite along with 28 wt% of PDMPD. The THR values of the PS/PDMPD composites were dropped by 12% and 9% in the presence of BP and ZnB, respectively [97]. Furthermore, the replacement of PDMPD with low amounts of BP or ZnB did not affect the char formation trend in the styrenic composite.

The influence of the FR chemical structure on its mechanism of action was extensively studied by Beach et al. (2008) [98]. According to the authors, PS with added HBCD and S-containing FR additives exhibited an enhanced degradation of the polymeric matrix, whereas PS with P-based structures such TPP or triphenylphosphine oxide (TPPO) had a minimal impact on the polymer degradation behaviour [98]. Interestingly, a combination of S with TPP resulted in much better fire-retarding performance owing to the synergism between S and P within the PS [98]. The fire-retarding performance of the ABS copolymer with different aryl cyclic phosphorous FRs revealed that the phosphonate compounds were more thermally stable and generated higher char yields as opposed to phosphinates [99]. There is a range of publications dedicated to synergistic formulations, for HIPS and ABS, containing red phosphorous combined with other additives: EG [100,101,102], Mg(OH)_2_ [103,104,105], and melamine polyphosphate [106]. 

In summary, the additive modifications of styrenic polymers with different P-containing FRs remain popular approaches to improving their fire performance. The analysis of the latest literature precedents shows that the majority of the P-containing additive FRs are incorporated into the polymer matrix, via the melt or the solution-blending techniques, at relatively high loadings. The evaluation of mechanical and physical properties proved that these levels of FRs, when added to the polymers, adversely affect the key properties, in particular, the impact and/or tensile strengths, and the density of the foams. In recent years, the multi-component hybrid formulations produced from organophosphorus compounds in combination with either transition metals, or with other inorganic compounds, were used successfully to tackle this problem by lowering the amounts of FRs to 2–10 wt%. Many researchers found that a combination of the gaseous and the condensed phase mechanisms are responsible for the activity of FRs in styrenic polymers after the additive modifications. In addition, intumescent fire-retardant actions of some compositions are also explored to passively protect some styrenic systems. Although the additive modifications of styrenic polymers became the simplest way to impart fire retardance, the dispersion and compatibility, recyclability, and toxicity of modified polymers are still questionable. In addition, developing the new P-containing additives based on inorganic/organic compounds is a cost-consuming task. In this context, the chemical incorporation of the FRs into the polymeric structure, which is discussed in the next section of the review, is believed to overcome these problems and provides some advantages over the additive modification approach.

### 4.2. Reactive Modifications of Styrenic Polymers

The chemical incorporation of fire-retarding species into the polymeric chains through covalent bonding is known as a reactive modification. The P-containing monomer(s) with double (-C=C-) bonds can be *co-* or *ter-* polymerised with the monomer of styrene (St) to produce the polymeric systems with improved fire retardance [48,107,108]. The reactive modification is particularly attractive because the fire-retarding groups, which are linked to the polymeric chains via covalent bonds, tend not to leach out from the polymer during its processing and the consequent use. In addition, the reactive FRs, which are directly integrated into the structures of polymers during their syntheses, are homogenously dispersed throughout the polymer on a molecular level, thus reducing their loadings required to become effective FRs. On the other hand, the lower amounts of fire-retarding groups less adversely affect the overall chemical, physical, and mechanical properties of the modified polymer, with the assurance of better fire retardance [1,5,35]. The reactive P-containing FRs are attractive not only as the halogen-free alternatives but also as more sustainable options, since the polymers with chemically linked modifications are more likely to be recycled compared to the multi-component materials with various additives. 

#### 4.2.1. Organic Phosphates and Phosphonates (P^+5^)

**Figure 17:**
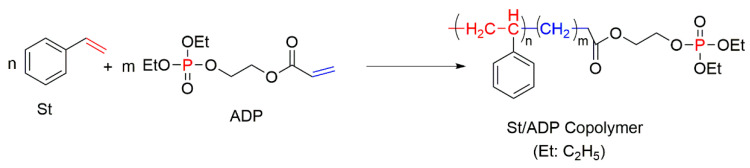
Free radical copolymerisation of styrene (St) with 2-(acryloyloxy)ethyldiethylphosphate (ADP) [109].

The *co-*polymerisation of St with P-containing unsaturated monomers (see Figure 1) can be an effective way to increase the thermal stability and fire retardance of styrenic polymers. There is a wide range of organic P-containing FRs available including phosphates, phosphonates, polyphosphonates, hybrid metal phosphonate salts, etc. [5,38,41,109,110,111,112,113,114,115,116,117,118,119,120,121,122,123,124,125]. The mechanism and the efficiency of FRs depend not only on the chemical structure of a P-bearing compound itself, but also on its interaction with the polymeric backbone during pyrolysis and combustion. The use of synergists also has an impact on the predominant mode of fire retardation [34,35]. Another factor affecting the fire retardance is the position of P-containing functionalisation within the polymer chain. For example, the free radical polymerisation of St with different P-based compounds such as phosphorylated acrylates and methacrylates, 2-(acryloyloxy)ethyldiethylphosphate (ADP), and phosphonamide (Figure 17) showed that only chain-end and chain-middle phosphorylation resulted in the improved thermal resistance and fire retardance [109].

**Figure 18:**
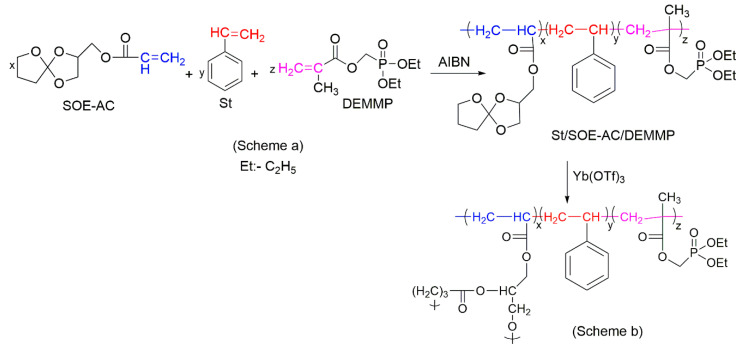
Schemes of *ter-*polymerisation of St and a ring opening of 1,4,6-trioxaspiro[4,4]-2-nonylmethyl acrylate (SOE-AC) moieties [110].

There are limited reports about the *ter-*polymerisation of St being employed as a route to improve the fire resistance of styrenic polymers. For example, Canadell et al. (2007) used diethyl(methacryloyloxymethyl)phosphonate (DEMMP) as well as 1,4,6-trioxaspiro[4,4]-2-nonylmethyl acrylate (SOE-AC) as the monomers (Figure 18, Scheme a) for the radical *ter-*polymerisation of St [110]. The obtained *ter*-polymer had higher LOI values of 25.2 vol% and higher thermal stability as indicated by the amounts of TG char residue raised by 18.4 wt% compared to the corresponding *co-*polymer of St with SOE-AC [110]. The presence of the cationic cross-linking agent, ytterbium triflate, Yb(OTf)_3_, initiated the double ring-opening of the pendant SOE moieties to develop a cross-linked network (Figure 18, Scheme b) that prevented the polymer from shrinkage and promoted the formation of an insulating protective layer by DEMMP fragments. Unfortunately, the authors of [110] did not consider the mechanism of fire retardance of the proposed formulations.

**Figure 19:**
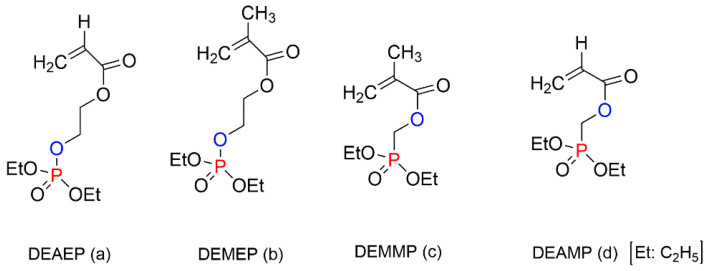
Chemical structures of unsaturated phosphates and phosphonates [38].

The impact of the chemical environment of the P atom on the efficiency and fire-retarding mechanism of reactively modified polymers was studied by Price et al. (2007) [38]. In this study, the influence of phosphate and phosphonate FRs on the fire behaviours of PS systems was compared. The reactively modified PS (with 3.5 wt% of P) contained the following monomeric units: diethyl(acryloyloxyethyl)phosphate (DEAEP), diethyl (methacryloyloxy ethyl) phosphate (DEMEP), diethyl(methacryloyloxymethyl)phosphonate (DEMMP), and diethyl (acryloyloxymethyl)phosphonate (DEAMP) (Figure 19a–d). The reactive modifications within the PS copolymers acted both in the condensed phase and in the gaseous phase. Moreover, the chemical environment of the P atom affected the mechanism of fire retardance to varying extents. During thermal decomposition, the copolymers with the integrated phosphates, DEAEP and DEMEP, formed phosphoric acid, while those with the phosphonate moieties, DEMMP and DEAMP, generated phosphonic acid in the condensed phase. These acidic species were believed to catalyse the Friedel–Crafts cross-linking of aromatic rings, thus increasing the thermal stability of the modified polymers. The stronger phosphoric acid was found to be promoting the cross-linking at a more rapid rate than the weaker phosphonic acid [38]. Thus, thermal degradation and the release of volatile products were much inhibited with phosphorus modification of the parent polymeric chains. For example, compared to the neat PS, the TTI value was doubled to 64 s for the styrenic copolymers with DEMEP fragments, whereas for the copolymer with DEMMP, only a 20 s increase in TTI was registered [38]. It was also reported that the acrylate derivatives exerted more impact on the condensed phase action than methacrylate comonomers, as the acrylates has a tendency to interfere with the H-transfer reactions occurring during the PS decomposition [38].

#### 4.2.2. DOPO Derivatives (P^+5^)

**Figure 20:**
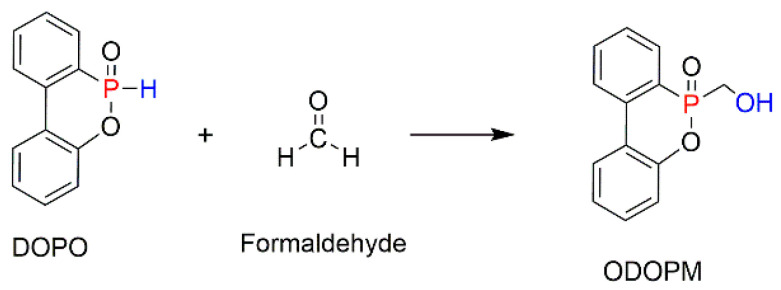
Synthesis of 2-(6-oxido-6H-dibenz[c,e][1,2]oxaphosphorin-6-yl)methanol (ODOPM) from DOPO [48,113,114].

Other commercially important P-containing FRs are based on DOPO or its derivatives prepared by the chemical transformation of the P–H bond within its molecule. These compounds have been employed as the additive modifications in a variety of polymeric materials [62,86,111,112]. However, as a relatively small molecule, the DOPO-based additive can be easily lost, thus limiting its application in styrenic polymers. An effective method to overcome this shortcoming is to modify the structure of DOPO with other known FRs or to incorporate them during polymerisation processes in the presence of other reactive monomers. The most commonly used method is the chemical modification of DOPO through the reaction with paraformaldehyde, which resulted in the formation of 2-(6-oxido-6H-dibenz[c,e][1,2]oxaphosphorin-6-yl)methanol (ODOPM), as shown in Figure 20 [48,113,114].

**Figure 21:**
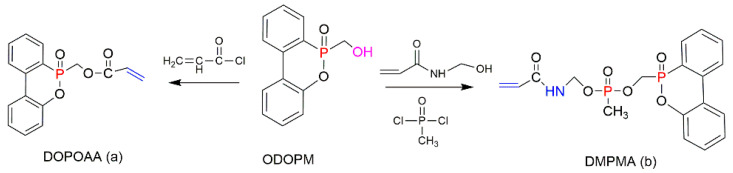
Syntheses of different DOPO derivatives from ODOPM precursor [48,113].

In several studies, ODOPM has been utilised as a precursor compound having a hydroxyl (OH) group that is able to undergo further chemical transformation (e.g., condensation reactions), producing different unsaturated compounds suitable for the reactive modifications [48,113,114]. For instance, Sun et al. (2020) functionalised ODOPM with acryloyl chloride, leading to the formation of DOPO–methylacrylate denoted as DOPOAA (Figure 21a) [113]. Then, this DOPO-bearing monomer was incorporated into the PS chains through the emulsion polymerisation. The contents of DOPOAA units integrated into the chains of the copolymer were found to be 3.30 and 5.97 wt%. The TGA results of both St/DOPOAA copolymers showed no significant increase in the values of T_i_, T_max_ and the amounts of a TG char formed. However, the maximum degradation rate (R_max_) was found to be reduced by 32% and 46% for the copolymers with 3.30 and 5.97 wt% DOPOAA, respectively [113]. This trend can possibly indicate better thermal stability of the reactively modified PS. However, the condensed phase activity was limited, as the productivity of char was not improved significantly and remained below 1 wt% as for the homopolymer. Meanwhile, the microcone calorimetric (MCC) measurements revealed that the PHRR values dropped significantly, from 972 W/g in the neat PS to 354 W/g, in the copolymer [113]. At the same time, the LOIs increased from 18 vol% for the PS to 26 vol% for the St/DOPOAA copolymer. These results confirmed the improved fire performance of PS with built-in DOPOAA monomeric units. It was also suggested that during thermal decomposition, the DOPO groups produced PO^●^ radicals, which entered the gaseous phase and eliminated the free radicals generated during combustion of the polymers [113].

**Figure 22:**
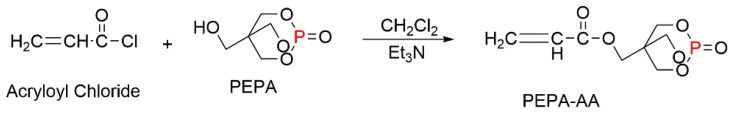
Synthesis route of 1-oxo-2,6,7-trioxa-1-phorsphabicyclo [2,2,2]oct-4-yl methyl acrylate (PEPA-AA) [114].

In another study, Yan et al. (2010) reactively modified PS with DOPOAA and with 1-oxo-2,6,7-trioxa-1-phorsphabicyclo [2,2,2]oct-4-yl methyl acrylate (PEPA-AA, Figure 22) [114]. The comparative studies proved that the PEPA-AA moieties were more efficient in promoting the condensed phase mechanism of fire retardation in the copolymer than the actions of the DOPO derivative. The results of the different flammability parameters of both copolymers are summarised in Table 1.

The TG char residue obtained in the inert atmosphere for the copolymer St/PEPAAA was increased to 36 wt%, while the residue from the St/DOPOAA was only 6.2 wt% under the same conditions. The lower char residue generated by the polymer with the covalently attached DOPO groups was explained by the formation of the weaker phosphonic acid instead of the stronger phosphoric acid during combustion [114]. The phosphonic acid seemed to be less effective in promoting acid-catalysed char production on the polymer surface. The similar trend was observed by Sun et al. (2020) [113]. Nevertheless, in the air atmosphere, the amount of char residue formed by the St/DOPOAA was increased up to 19.3 wt%. This confirmed that the oxygen-containing atmosphere had an accelerating effect on the char formation by DOPO groups, since the phosphonic acid is oxidised to phosphoric acid, which works as an excellent char-forming agent. Meanwhile, during the thermal oxidative degradation of the St/DOPOAA polymer, the cross-linking of phosphate structures increased [113].

**Figure 23:**
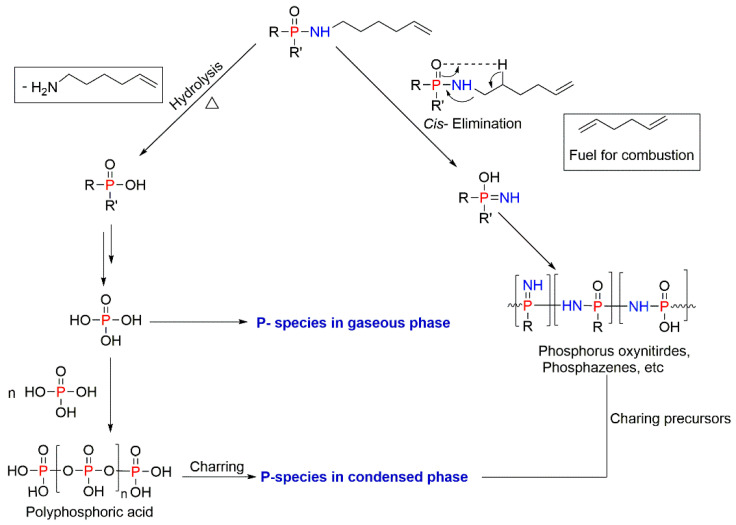
Decomposition mechanisms of the reactive P–N-containing FRs [116].

Recently, the use of synergistic reactive FRs with P- and/or N-containing moieties has gained great attention owing to their effectiveness and lower negative impact on the environment [115]. The P–N synergism can accelerate the phosphorylation of the polymer, during thermal decomposition/combustion, by enhancing the in situ production of phosphoric acid due to catalysing *cis*-elimination (Figure 23) [90,116]. This reaction retains phosphorus in the condensed phase, thereby promoting a char formation and thermal stabilisation of the polymeric substrate [116]. The *cis*-elimination process resulted in the formation of different compounds containing P–N linkages, thus enabling the higher retention of phosphorus in the condensed phase and more enhanced char formation during decomposition [116]. As the content of P in the char increased, the flame inhibition slowed, since the phosphorus in the condensed phase competed with the P-species release to the gaseous phase [116,117].

**Figure 24:**
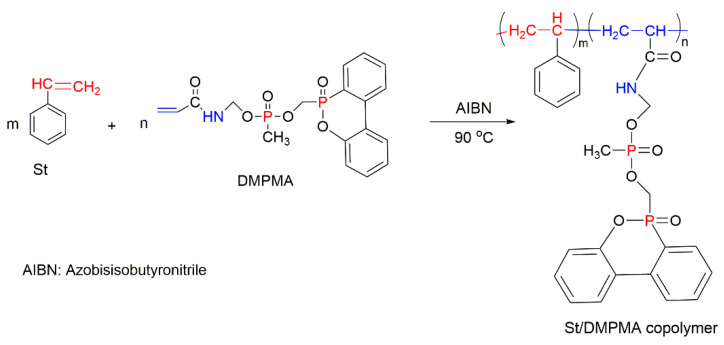
Copolymerisation of St with 2-[(6-oxido-6H-dibenzo[c,e][1,2]oxaphosphinin-6-yl)-methoxy]methyl phosphate N-methyl acrylamide (DMPMA) initiated by azobisisobutyronitrile (AIBN) [48].

The nucleophilic substitution of the above-mentioned ODOPM with *N*-hydroxymethylacrylamide and methyldichlorophosphate is behind the synthesis of novel P–N-containing reactive monomer 2-[(6-oxido-6H-dibenzo[c,e][1,2]oxaphosphinin-6-yl)-methoxy]methyl phosphate *N*-methyl acrylamide (DMPMA) (Figure 21b) [48]. The free radical bulk polymerisation of St with DMPMA resulted in the copolymer having better fire retardance (Figure 24) than the homopolymer [48]. Indeed, the MCC measurements showed that the values of the PHRR were decreased from 840 W/g for the unmodified PS to 360 W/g for the copolymer containing 10 wt% of the incorporated DMPMA units. The combined effect of the condensed phase action and the gaseous phase activity in modified PS symbolised the synergistic effect of P- and N-bearing groups [48]. Meanwhile, the toxicity studies (with the aid of a steady-state tube furnace) established that the copolymers of St with DMPMA produced more CO and had higher smoke density compared to the same parameters measured for the neat PS [48].

**Figure 25:**
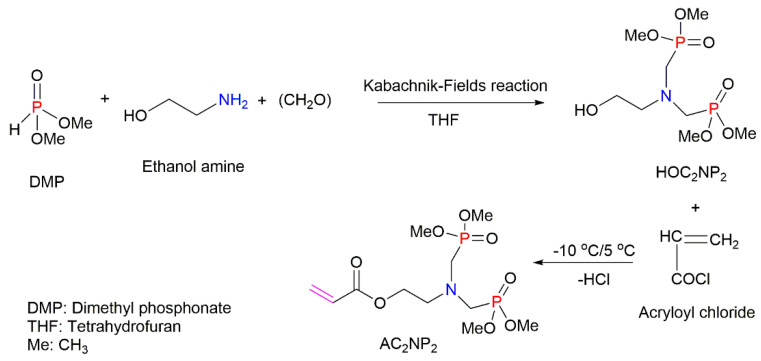
The Kabachnik–Fields reaction to synthesise ethyl-*N,N*-tetramethyl-bis(phosphonate)-bis(methylene)amine acrylate (AC_2_NP_2_) [107,119].

Wendels et al. (2017) reviewed the available synthetic methods to design new organophosphorus FRs [118]. One example is the Kabachnik–Fields reaction involving three components with carbonyl and amino groups coupled with dialkyl phosphonate (Figure 25). This reaction can convert the P–H bond in the organophosphorus compound to the P–C bond, forming α-aminophosphonates [118]. The P–N-containing compound (HOC_2_NP_2_), prepared through the Kabachnik–Fields process, can react further with acryloyl chloride to obtain ethyl-*N,N*-tetramethyl-bis(phosphonate)-bis(methylene)amine acrylate (AC_2_NP_2_) [119]. The solution polymerisation of St with AC_2_NP_2_, at 65 °C, using dioxane as a solvent, resulted in the formation of the polymer with enhanced fire performance. The flammability studies indicated the increase in the thermal stability of the copolymers with 10–25 wt% of AC_2_NP_2_ monomeric units. The PHRR values, measured by MCC, in the chemically modified PS were significantly reduced and dependent on the AC_2_NP_2_ content. The PHRR was dropped by 36%, 41%, and 42% in the styrenic copolymers with 10 wt%, 20 wt%, and 25 wt% of AC_2_NP_2_, respectively [119]. However, the copolymer with 30 wt% of AC_2_NP_2_ had the lower values of T_i_ and T_max_, the higher values of PHRR, and the greater mean mass loss rate (MMLR) compared with those measured in other copolymers. These results revealed that when the amount of the FR incorporated into the polymer exceeds a certain limit, it does not necessarily improve its fire performance, but it may have a negative impact on its properties. Meanwhile, the St/AC_2_NP_2_ copolymers exhibited higher TG char residue under the air atmosphere than that formed under the inert conditions. For example, the copolymer with 10 wt% of AC_2_NP_2_ had nearly 10.00 wt% of char residues in the air atmosphere, at 500 °C, whereas under the nitrogen atmosphere, the char residue was only 2.05 wt%. This indicated an influence of oxygenated atmosphere on the char formation and thermal stability of P-containing styrenic copolymers [119].

**Figure 26:**
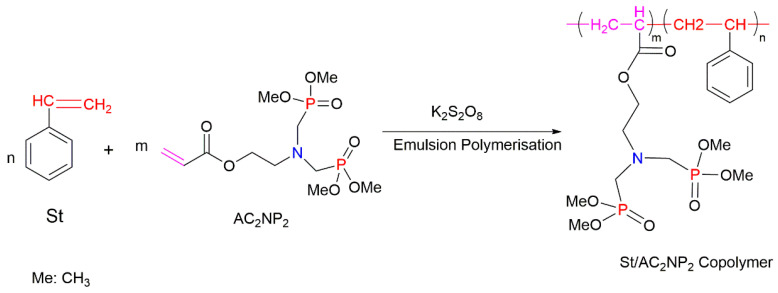
Emulsion polymerisation of St with AC_2_NP_2_ [107].

In another study, Cui et al. (2017) used the same P–N comonomer, AC_2_NP_2_, in the amounts ranging from 5 to 20 g for the preparation of styrene-based copolymer via the seeded emulsion polymerisation (Figure 26) [107]. During the emulsion polymerisation, water was used as the emulsion medium along with the emulsifiers, sodium lauryl benzene sulfate and sodium *p-*styrene sulfonate. The fire-retarding mechanism of the AC_2_NP_2_ units was determined from various characterisation and flammability studies, clearly highlighting the synergistic P–N effects. The impact of this synergism strengthened as the content of AC_2_NP_2_ in the copolymer grew up to 20 g [107].

Table 2 summarises the data obtained from various flammability studies of the PS homopolymer and the copolymer of St with 10 wt% of AC_2_NP_2_ prepared through the solution and emulsion polymerisation techniques [107,119]. The polymeric samples prepared in the solution or emulsion showed higher char residue (CR) under the air atmosphere than that produced in the inert atmosphere. Similarly, other thermal and fire-retarding properties of the polymers have been increased after the chemical incorporation of AC_2_NP_2_. Moreover, the analysis of the flammability data indicated that the emulsion polymerised styrenic materials had better fire-retarding efficiency than that of the polymers prepared in solution. Flammability studies revealed that during heating, advanced pyrolysis of the St/AC_2_NP_2_ copolymer occurred due to the breakdown of the phosphate bond, thus leading to the dehydration and degradation of AC_2_NP_2_ [107]. The phosphoric acid generated by AC_2_NP_2_ amounts at high temperatures produced polyphosphoric acid and polyphosphate, which further participated in the formation of protective layers, boosting the condensed phase activity of FR groups. These layers prevented the release and diffusion of flammable gases. The authors also pointed out at the gaseous phase the fire-retarding effects of the AC_2_NP_2_ groups [107].

**Figure 27:**
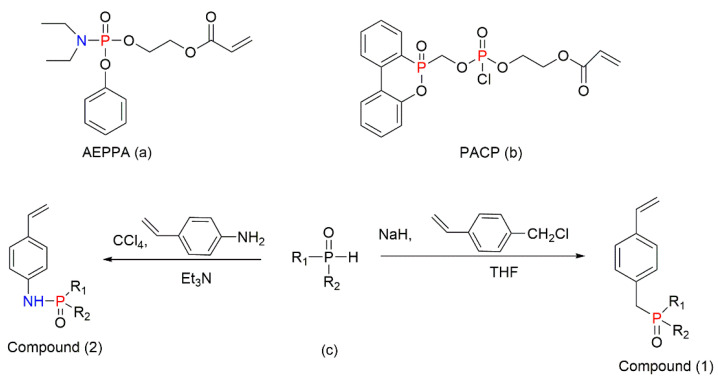
A chemical structure of acryloxyethylphenoxy phosphorodiethylamidate (AEPPA) (**a**) and 2-((6-oxidodibenzo[c,e][1,2]oxaphosphinin-6-yl)methoxy)acryloxyethyl)chlorophosphate (PACP) (**b**); syntheses of styrene-based reactive FRs (**c**) [120,122,123].

Another novel monomer with the structure shown in Figure 27a, acryloxyethylphenoxy phosphorodiethylamidate (AEPPA), was synthesised by the esterification reaction of phenyldichlorophosphate (PDCP) with hydroxyethyl acrylate (HEA), followed by the reaction with diethylamine [120]. The AEPPA monomer was incorporated into the styrenic chains through the bulk polymerisation technique in a two-step process. In the first step, polymerisation was carried out with AEPPA and St in the presence of AIBN at 90 °C. Later, the obtained viscous mixture was moulded at 80 °C for 16 h. The flammability studies showed that the incorporation of 10 wt% of AEPPA increased the char residue from 1.49 wt% in PS to 7.00 wt% in the St/AEPPA copolymer. Similarly, the introduction of AEPPA units into the PS chains considerably reduced the release of combustible compounds evolved and cooled the pyrolysis zone by diluting the hot atmosphere with non-flammable products such as CO_2_, NH_3_, N_2_, and P-based compounds produced by the AEPPA [121]. Meanwhile, the incorporation of AEPPA in the amounts higher than 15 wt% had no significant impact on the fire-retarding performance of the copolymer [120].

The synergistic P–N effect on the flammability of styrenic polymers was found in a comparative study carried out by Dumitrascu and Howell (2012) [122]. They prepared two styrenic copolymers, one containing only P atoms (Compound (**1**), Figure 27c), and the other containing both P and N atoms, joined via the P–N bond, (Compound (**2**), Figure 27c). The TGA data suggested that the presence of the P–N-bearing units enhanced the impact of P on the thermal stability of PS in terms of the increased value of T_i_ during thermal degradation. The polymer with the integrated moieties of Compound (**1**) had the T_i_ of 416 °C, while in the copolymer containing monomeric units of Compound (**2**), these temperature values were about 10 °C higher. The synergistic P–N effect, leading to the boost of the condensed phase activity, was evident from the higher char yields at 600 °C: 16.3 wt% of the residue was obtained for the polymeric material containing units of Compound (**1**), and 26.2 wt% of the residue was obtained for the copolymer with the groups of Compound (**2**) [122]. Similarly, the copolymers with P–N-containing moieties displayed reduced PHRR values, measured in MCC, and better fire-retarding performance compared to those containing only P-containing units. Thus, all the studies discussed above clearly demonstrated the benefits of utilising the P–N synergism and its positive impact on the thermal stability and fire retardance emanating from the incorporation of P–N-containing comonomers into the PS backbone.

#### 4.2.3. Inorganic FRs Modified with Organophosphorus Compounds (P^+5^)

The reactive modification of styrenic polymers with the inorganic P-containing compounds remains rare, which is possibly due to the difficulties associated with the chemical methods of incorporating the FRs into the polymeric chains, i.e., a lack of polymerisable double bonds. Nevertheless, the covalent functionalisation of some inorganics with unsaturated organophosphorus compounds provided an opportunity to achieve this. The use of graphene oxide (GO) as a FR additive was already discussed in this review earlier [50,82,83]. Recently, Dai et al. (2018) covalently functionalised GO with a novel organic P-containing compound, 2-((6-oxidodibenzo[c,e][1,2]oxaphosphinin-6-yl)methoxy)acryloxyethyl)chlorophosphate (PACP), the structure of which is shown in Figure 27b [123]. From 1 to 3 wt% of the FGO were incorporated into the PS chain via the bulk polymerisation method, using benzoyl peroxide as the initiator and heating the reaction mixture to 90 °C. The good dispersion of the FGO within the polymer and the strong interfacial bonds between the FGO and the polymer matrix resulted in the significant improvement of thermal behaviour and fire resistance [123]. The TG char residue of the copolymer with 3 wt% of reactive FR increased from 0.33 to 6.50 wt% under the inert atmosphere. At the same time, the heat release capacity (HRC) of the same copolymer decreased dramatically, from 984 J/(g∙K) for the unmodified PS to 599 J/(g∙K) for the copolymer [123]. These studies also demonstrated the combined effect of the gaseous and condensed phase activities mainly originating from the FGO moieties by the inhibition of combustion chain reactions and catalysing the char formation, respectively [123].

In another study, the condensation reaction between the carboxyl group on the surface of GO and the amino group of *N*-aminoethylpiperazine (AEPZ) resulted in the formation of AEPZ-GO [124]. Then, the AEPZ-GO was reacted with the phosphonate compound, 3,9-bis(chloro)-2,4,8,10-tetraoxa-3,9-diphosphaspiro[5.5]undecane-3,9-dioxide, to prepare a hyper-branched FR. In this case, the FGO was prepared by grafting the hyper-branched FR onto the surface of the GO via the Michael addition reaction [124]. The PS–FGO composites were prepared by in situ polymerisation, starting with a very low amounts: 0.1 wt%, followed by 0.5 wt%, 1.0 wt%, and 2.0 wt% of the FGO. The MCC results showed that the PHRR values decreased from 840 W/g for the unmodified polymer to 514 W/g by the incorporation of 2 wt% of the FGO in the polystyrene chains. Meanwhile, the char yield, obtained from TGA under the air atmosphere, was increased from 0.5 wt% in the neat PS to 6.5 wt% in the PS-FGO [124]. In addition, other fire hazards were brought under control, for example by reducing the CO and CO_2_ emissions. The enhancement in the fire retardance was attributed, partially, to the uniform dispersion of the FGO layers within the PS matrix. These layers were able to capture oxygen free radicals in the gaseous phase and inhibit the degradation of the polymer chains. The decomposition of the hyper-branched FR also promoted the char formation on the surface of the burning material, thereby reinforcing the barrier effect of the FGO sheets [124].

Another inorganic compound, α-zirconium phosphate, α-Zr(HPO_4_)_2_∙H_2_O (labelled as α-ZrP) was used along with the P–N-containing FR, AEPPA (Figure 27a) to prepare the copolymer by the bulk polymerisation [125]. The comparative MCC studies of different polymeric samples showed that the incorporation of 3 wt% of α-ZrP and 10 wt% of AEPPA significantly reduced the HRC from 915 J/g∙K for the unmodified polymer to 563 J/g∙K for the copolymer, while the THR values dropped from 39.0 to 36.6 kJ/g. Similarly, the char residue from the St/AEPPA copolymer was almost doubled after the incorporation of α-ZrP into the St/AEPPA copolymer. It was estimated that the enhanced fire retardance was attributed to the increased char formation and decreased mass loss rate [125].

Clearly, the reactive route of modification enabled the reduction in the amounts of FRs required to achieve the sufficient fire-retarding effects when compared to the additive modifications. However, it is questionable whether these reductions were reasonable, since most of the studies on average utilised 10–25 wt% of FRs. These issues can be solved by employing the P–N synergism in reactive modifications, although this approach is not systematically explored to date. Additionally, the effects of these modifications on the physical and mechanical properties, as well as on the toxicity of fire effluent, were not carried out in many studies; thus, we do not wish to provide here our hypothetical views. Nevertheless, the reactive modification allowed the phosphorus to be uniformly dispersed in the polymer matrix, thus promoting better charring and enhancing the condensed phase of fire retardation. Meanwhile, in the case of additive modifications, there is a possibility that phosphorus FRs may leave the parent polymer, for example at lower temperatures, hence the interactions with decomposition pathways of the polymer remain limited.

## 5. Conclusions

From the current review of reactive and additive modifications of styrenic polymers with P-containing compounds, it is possible to draw some general conclusions about their efficiency as fire retardants. Owing to the presence of chemically reactive aromatic rings, polystyrene chains are subjected to varying degrees of cross-linking reactions during post-polymerisation modifications. Whilst the *co-*polymerisation reaction might provide a more technologically attractive alternative, the choice of a suitable comonomer often becomes a limiting factor, especially in the large-scale preparations. Therefore, the additive route is generally considered as more viable from a commercial point of view. However, this route is often associated with relatively high loadings to achieve an acceptable degree of fire retardance to styrenic polymers.

The use of the additive P-containing fire retardants remains very popular due to the simplicity of this approach. Many studies carried out in recent years were focussed on varying the ratios of the components in the additive FRs, on the incorporation of the nanomaterials with varied chemical constitutions and of different origins, and then on monitoring the thermal parameters (decomposition temperature, heat release rates, char yields, etc.). Even though these studies have demonstrated an increase in the overall fire-retardant properties, as identified through some prescriptive laboratory-scale techniques/tests, they still remain predominantly in the realm of an academic pursuit. Some compositions such as graphene-based ones are rather complex and expensive; their poor compatibility and the unknown environmental and health impacts prevent their wider industrial use.

The problems associated with high loadings of additive FRs can be reasonably solved by adopting the reactive modifications or through the use of synergists, which are frequently combined with nanoclays, carbon nanotubes, or graphene/graphite compositions. However, only a limited number of publications reported the successful suppression of smoke or toxic gases released during burning of the modified styrenic products. From a practical point of view, there are some other challenges that need due consideration while formulating any FR strategy, including sustainability, cost-effectiveness, and the ease of processing, and hence the commercial viability of the proposed formulations; the environmental implications of the nanofillers, additives, or monomers; the overall efficacy of the modifying compounds in the case of a fire; etc. None of the reviewed publications had systematically studied the toxicity of the gaseous products in the fire effluents of the modified styrenic compositions. As the use of P-containing FRs becomes more widespread, their impact on public health and the toxicity of their decomposed fragments should be the key tasks for the researchers in the near future.

There are also some serious concerns related to a loss of FRs during the life cycle of styrenic products, potentially causing harm to the environment and negatively affecting human health. The future trends will likely involve the greater use of bio-inspired fire-retardant solutions or P-containing waste materials [126,127,128,129]. However, only a small number of bio-based P-containing formulations were proposed recently, and they were required at very high loadings [127,128].

The effectiveness of phosphorus-based compounds depends on various parameters such as the P-content present in the polymeric material, the chemical environment of the P atom, its position in the polymeric chain, the oxidation state, and the distribution of the modification within a host polymeric matrix. The chemical nature of the groups covalently attached to the phosphorus atom also greatly influences the fire-retarding properties of P-bearing FRs. Generally, phenyl or aromatic groups bonded to the P atom could assist in maintaining the higher thermal stability of the fire-retarded styrenic polymers. The comparative study of PS with phosphorus compounds containing various pendant groups revealed that those with diphenyl phosphonates and phosphates had much higher thermal stability compared to the materials with the incorporated diethyl phosphorus compounds [130].

The mode of action, which is essential for an adequate FR selection, varies from one formulation to another and depends on several factors. Given a complex nature of chemical components and mechanistic pathways that underpin the combustion cycle of a polymeric material, in general, and the relative predominance of condensed-, gaseous- and inter-phase, in particular, it is not often easy to identify the underlying physico-chemical processes unambiguously. Phosphorus compounds could function either as char promoters in the condensed phase or as fire inhibitors in the gaseous phase. The majority of the P-containing formulations were found to rely on a combination of both mechanisms. For the styrenic materials, the increasing of char content during combustion would be an important contribution to the improved fire retardance. The oxidation state of the phosphorus atom in a FR compound may influence the char-forming ability and the overall thermal stability of the parent polymer matrix. In general, the higher oxidation state of the phosphorus atom, as in the phosphate-based compounds, enables more efficient char production and improves the condensed phase activity of FR [34]. However, this trend can also vary widely, and often depends strongly on other factors including the chemical nature and constitution of the parent polymer matrix and modifying groups. Thus, more scientific data are required to draw conclusions about the effect of the oxidation number of P on the fire performance of the modified styrenic polymers.

The synergistic phenomena between P and several other elements have been postulated by some authors. Normally, synergism is taken into account when the fire retardance of a polymer is improved by the combined effect of P and another element in the comparison to using each element separately, at the same loading levels. Among various synergistic systems explored so far, the P–N synergism was shown to be very beneficial for PS, though it is not systematically studied and clearly explained. The *ter-*polymerisation of styrene with two different monomers containing P and N atoms is another important route to improve the fire-retarding properties of PS. However, it is also a less studied approach and could be considered as an upcoming and promising technique. The analysis of publications in the present review revealed a variety of analytical techniques and test methods employed to assess the fire retardance of styrenic polymers. In the majority of studies, the evaluation of fire retardance was confined to TGA, LOI, CC, MCC, UL-94 and Gas Chromatography-Mass Spectrometry (GC-MS). Thus, it would be very beneficial for researchers to explore the capabilities of ‘hyphenated’ techniques such as TGA-MS, TGA-FTIR, laser pyrolysis/time of flight-mass spectrometry (LP/TOF-MS) and a steady-state tube furnace test, which would provide a clearer picture about the mechanisms of fire retardance. In addition, there is a need for researchers to adapt other characterisation techniques such as rheometry or Dynamic Mechanical Analysis (DMA) to determine the important attributes, which would help to forecast the behaviour of fire retarded polymers in real-scale fire scenarios.

## Figures and Tables

**Table 1:** Comparative data of PS/PEPA-AA and PS/DOPOAA (DOPO–methylacrylate) copolymers from fire tests [114]. LOI: limiting oxygen index, TGA: thermogravimetric analysis, CR: char residue.

Copolymer	UL-94 Rating	Dripping	LOI	^1^ CR (wt%) (N_2_/Air)
St/PEPAAA	V-0	No	27.0	36.0/34.6
St/DOPOAA	V-2	Yes	24.2	6.2/19.3

^1^ Char residue obtained in TGA runs at 500 °C.

**Table 2:** Flammability data for PS and St/AC_2_NP_2_ copolymer prepared by different polymerisation techniques [107,119]. MCC: microcone calorimetry, PHRR: peak heat release rate.

Polymer	Polymerisation Technique	T_i_ (°C) (N_2_/Air)	T_max_ (°C) (N_2_/Air)	PHRR’ (W/g)	PHRR” (kW/m^2^)	CR (wt%) (N_2_/Air)	LOI (vol%)
Neat PS	Solution	392/321	418/377	751	-	0.25/1.50	18.5
St/AC_2_NP_2_	Solution	385/363	423/398	480	-	2.05/9.77	21.0
Neat PS	Emulsion	403/319	428/381	-	1283	1.94/0.33	18.0
St/AC_2_NP_2_	Emulsion	418/321	447/413	-	824	5.89/6.14	22.0

PHRR’: measured using MCC. PHRR”: measured using CC. CR: char residue formed at 550 °C.
